# An engineered ACE2 decoy neutralizes the SARS-CoV-2 Omicron variant and confers protection against infection in vivo

**DOI:** 10.1126/scitranslmed.abn7737

**Published:** 2022-04-26

**Authors:** Nariko Ikemura, Shunta Taminishi, Tohru Inaba, Takao Arimori, Daisuke Motooka, Kazutaka Katoh, Yuhei Kirita, Yusuke Higuchi, Songling Li, Tatsuya Suzuki, Yumi Itoh, Yuki Ozaki, Shota Nakamura, Satoaki Matoba, Daron M Standley, Toru Okamoto, Junichi Takagi, Atsushi Hoshino

**Affiliations:** ^1^ Department of Cardiovascular Medicine, Graduate School of Medical Science, Kyoto Prefectural University of Medicine, Kyoto 602-8566, Japan.; ^2^ Department of Infection Control and Molecular Laboratory Medicine, Graduate School of Medical Science, Kyoto Prefectural University of Medicine, Kyoto 602-8566, Japan.; ^3^ Laboratory for Protein Synthesis and Expression, Institute for Protein Research, Osaka University, Osaka 565-0871, Japan.; ^4^ Department of Infection Metagenomics, Research Institute for Microbial Diseases, Osaka University, Osaka 565-0871, Japan.; ^5^ Integrated Frontier Research for Medical Science Division, Institute for Open and Transdisciplinary Research Initiatives (OTRI), Osaka University, Osaka 565-0871, Japan.; ^6^ Department of Genome Informatics, Research Institute for Microbial Diseases, Osaka University, Osaka 565-0871, Japan.; ^7^ Department of Systems Immunology, Immunology Frontier Research Center (IFReC), Osaka University, Osaka 565-0871, Japan.; ^8^ Department of Nephrology, Graduate School of Medical Science, Kyoto Prefectural University of Medicine, Kyoto 602-8566, Japan.; ^9^ Institute for Advanced Co-Creation Studies, Research Institute for Microbial Diseases, Osaka University, Osaka 565-0871, Japan.; ^10^ Center for Infectious Disease Education and Research (CiDER), Osaka University, Osaka 565-0871, Japan.

## Abstract

The Omicron (B.1.1.529) SARS-CoV-2 variant contains an unusually high number of mutations in the spike protein, raising concerns of escape from vaccines, convalescent serum and therapeutic drugs. Here we analyzed the degree to which Omicron pseudovirus evades neutralization by serum or therapeutic antibodies. Serum samples obtained 3 months after two doses of BNT162b2 vaccination exhibited 18-fold lower neutralization titers against Omicron than parental virus. Convalescent serum samples from individuals infected with the Alpha and Delta variants allowed similar frequencies of Omicron breakthrough infections. Domain-wise analysis using chimeric spike proteins revealed that this efficient evasion was primarily achieved by mutations clustered in the receptor-binding domain, but that multiple mutations in the N-terminal domain contributed as well. Omicron escaped a therapeutic cocktail of imdevimab and casirivimab, whereas sotrovimab, which targets a conserved region to avoid viral mutation, remains effective. Angiotensin-converting enzyme 2 (ACE2) decoys are another virus-neutralizing drug modality that are free, at least in theory, from complete escape. Deep mutational analysis demonstrated that, indeed, an engineered ACE2 molecule prevented escape for each single-residue mutation in the receptor-binding domain, similar to immunized serum. Engineered ACE2 neutralized Omicron comparably to the Wuhan strain and also showed a therapeutic effect against Omicron infection in hamsters and human ACE2 transgenic mice. Like previous SARS-CoV-2 variants, some sarbecoviruses showed high sensitivity against engineered ACE2, confirming the therapeutic value against diverse variants, including those that are yet to emerge.

## Teaser

The SARS-CoV-2 Omicron variant exhibits effective evasion from antibodies but remains highly susceptible to blockade by engineered ACE2.

## INTRODUCTION

The severe acute respiratory syndrome coronavirus 2 (SARS-CoV-2) variant, B.1.1.529, was detected in Botswana on November 11^th^, 2021 and spread rapidly and globally. On November 26^th^, the World Health Organization (WHO) classified B.1.1.529 as the Omicron variant of concern (VOC). Omicron possesses 26 to 32 mutations, 3 deletions and one insertion in the spike protein. Among these, 15 mutations are located in the receptor-binding domain (RBD). Spike protein mutations have the potential to enhance transmissibility, enable immune evasion, or both (*1*). Compared to previous variants, Omicron contains far more mutations in the spike protein, and such mutations are expected to dramatically alter the characteristics of SARS-CoV-2. According to routine surveillance data from South Africa, Omicron has higher transmission and risk of reinfection due to immune evasion. In addition, multiple mutations in the RBD have been reported to impact escape from therapeutic monoclonal antibodies, even in cocktail regimens (*2*–*4*).

We previously developed an engineered ACE2 containing mutations to enhance affinity toward SARS-CoV-2 spike protein that showed virus-neutralizing capacity comparable to therapeutic monoclonal antibodies (*5*). The advantage of the ACE2-based decoy is its resistance to virus escape mutations. Mutant spike proteins escaping from ACE2 decoy may appear, but they would have limited binding affinity toward the native ACE2 receptors on host cells, making such resultant viruses unfit to propagate due to reduced or even absent infectivity. In fact, engineered ACE2 successfully neutralized previous viral variants as well as SARS-CoV-1 and showed no signs of vulnerability to escape mutants when added at suboptimal concentration during long-term culture (*5*).

Here, we examined the antigenic alterations of the Omicron variant and demonstrate that Omicron does indeed evade neutralization by vaccinated and convalescent serum samples. The ability of Omicron to escape was primarily due to mutations in the RBD, although those in the N-terminal domain (NTD) also contributed to some extent. A broad range of antibodies failed to neutralize Omicron, including a cocktail of imdevimab and casirivimab. However, the ACE2 decoy remained effective to Omicron, as well as to other sarbecoviruses.

## RESULTS

### Omicron emerged with numerous mutations in the spike proteins.

**Fig. 1.**
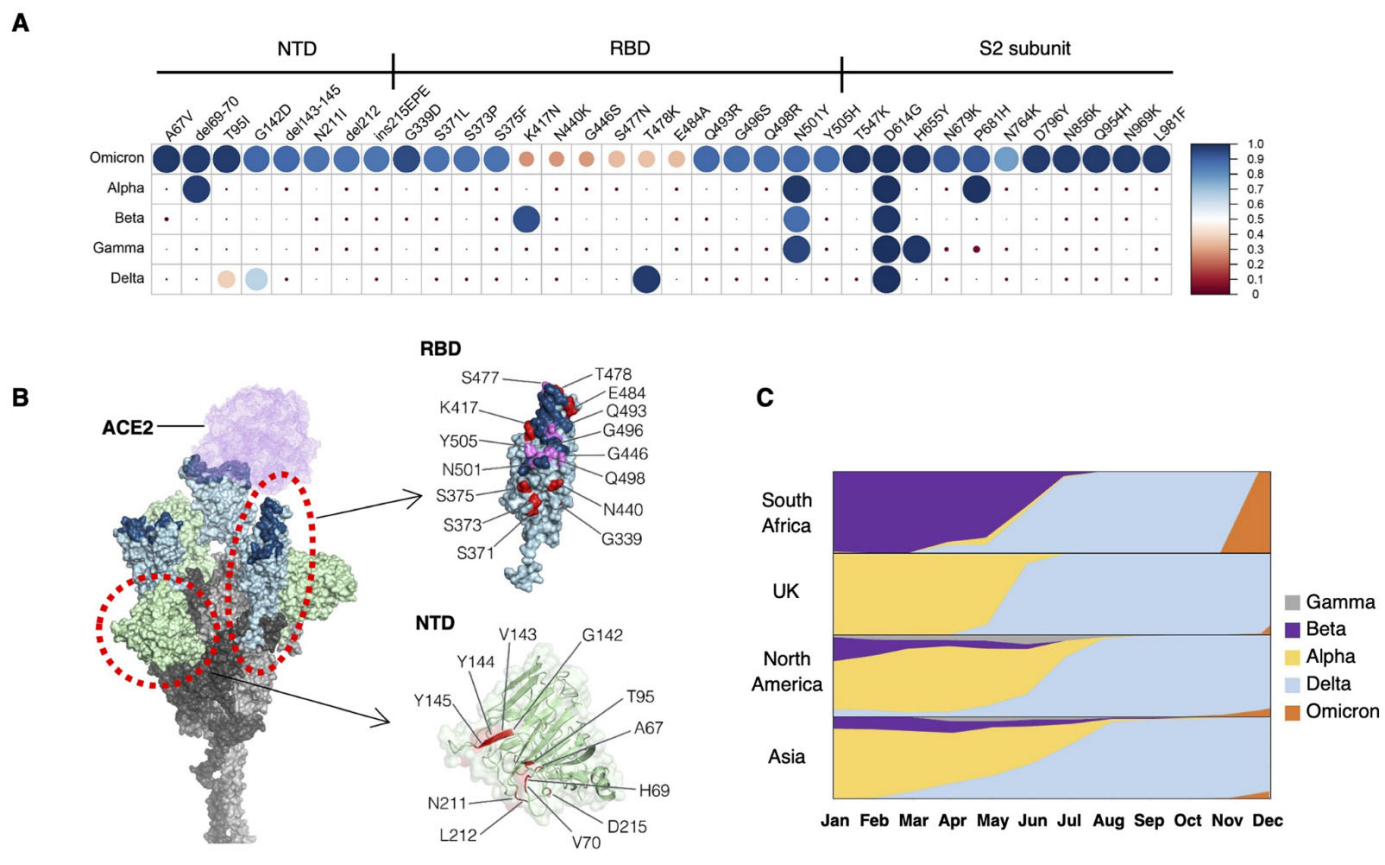
The Omicron (B.1.1.529) variant is characterized by the cluster of mutations in the spike protein. (**A**) Amino acid mutations in the spike protein are shown for the indicated SARS-CoV-2 strains. Mutation frequencies of each amino acid substitution in the spike protein were calculated using SARS-CoV-2 sequences reported in GISAID as 17^th^ Dec 2021 (Alpha = 1,138,704; Beta = 40,135; Gamma = 117,200; Delta = 3,441,137, and Omicron = 5,469 sequences). The circle size and color represent the mutation frequency according to the scale shown at the right. (**B**) A structural model of the SARS-CoV-2 spike trimer with all three RBDs in the open state based on PDB: 7A89 is shown. In the RBD, the RBM is highlighted in dark blue, and Omicron mutations are highlighted in purple or red in or outside the RBM. (**C**) The relative proportions of variant cases during 2021 in South Africa, United Kingdom, North America, and Asia are shown.

We first compared greater than 30 amino acids mutated in the Omicron variant of SARS-CoV-2 to other VOCs reported in the Global Initiative on Sharing Avian Influenza Data (GISAID) database. We observed that many mutations found in the Omicron variant were present in previous VOCs as a minor population (Fig. 1A). About 70% of mutations were located in the S1 subunit harboring the NTD and RBD. The NTD contains 4 missense mutations, 3 deletions, and 1 insertion, and most of these changes reside in the flexible loop region that is known to be the target of NTD-directed neutralizing antibodies (*6*). In the RBD, 10 of 15 missense mutations cluster in the receptor binding motif (RBM) (Fig. 1B). These characteristics suggest that mutations in Omicron are likely to influence the binding affinity of neutralizing antibodies, host receptors such ACE2, or both. The time course of dissemination of SARS-CoV-2 VOCs throughout world demonstrated that the Delta variant was rapidly replaced with the Omicron variant in South Africa (Fig. 1C). These data support the notion that mutations in the Omicron spike protein may enhance infection rates.

### Omicron evades vaccine and convalescent serum through mutations in the RBD and NTD.

**Fig. 2.**
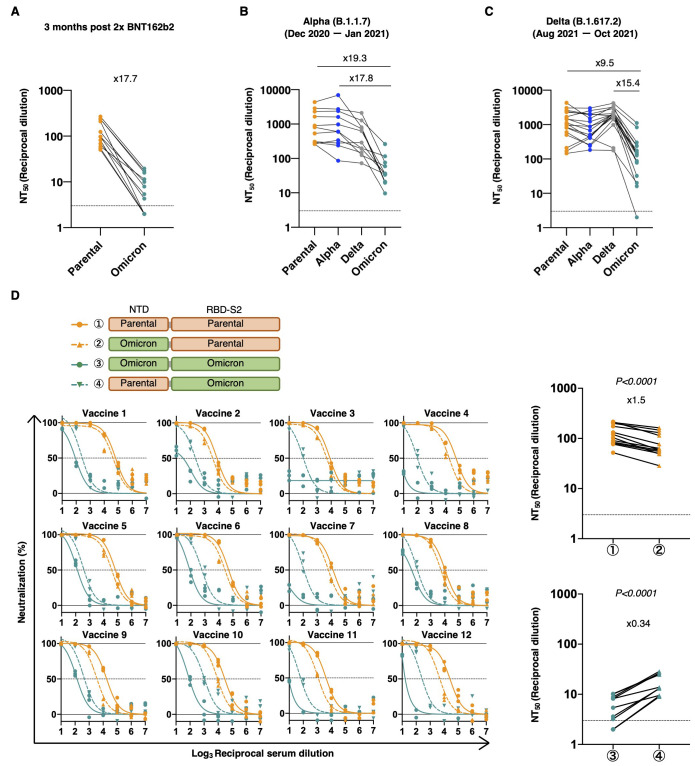
The Omicron variant evades immunity due to mutations localized not only in the RBD but also in the NTD. (**A to C**) Reciprocal dilution of 50% neutralization titer (NT_50_) values were determined in 293T/ACE2 cells for serum samples isolated from 12 individuals 3 months after two doses of BNT162b2 vaccination (A), convalescent serum from 11 patients infected with the Alpha variant (B), and 18 patients infected with the Delta variant (C). (**D**) Neutralization efficacy in 293T/ACE2 cells is shown for 12 vaccinated serum samples against parental virus (D614G mutation), parental virus with replacement with Omicron’s NTD, Omicron, and Omicron with replacement with parental NTD. The NT_50_ values are summarized in the right panels. n = 3 technical replicates. *P*-values were determined by two-sided paired *t*-tests. Dotted lines indicate detection limit.

To evaluate the infectivity of Omicron, we generated a pseudotyped virus harboring the spike protein of Omicron and assessed neutralizing activity of serum from BNT162b2-vaccinated or convalescent individuals against it. Virus neutralization assays with serum samples obtained from 12 individuals at 3 months after vaccination with two doses of the Pfizer-BioNTech vaccine BNT162b2 showed 17.7-fold lower neutralization titers against Omicron than the D614G mutation of the parental virus (Fig. 2A and fig. S1). We also collected convalescent serum samples before or after the Delta variant wave. Convalescent serum from individuals infected before the Delta variant wave (December 2020 through January 2021) showed 19.3 and 17.8-fold reduction compared with parental virus or Alpha. On the other hand, serum samples collected from individuals infected during or after the Delta variant wave (August 2021 through October 2021) exhibited 9.5 and 15.4-fold reduction compared with the parental virus or Delta, respectively. (Fig. 2B and C, fig. S1).

To examine the contribution of NTD mutations, we made a pseudovirus harboring a chimeric spike protein that contained Omicron’s NTD in the parental virus spike protein or the parental NTD in the Omicron variant spike protein. Replacing the parental virus NTD with the Omicron variant NTD mildly attenuated the neutralizing effect of vaccinated serum samples. In contrast, the removal of NTD mutations from Omicron increased susceptibility to vaccine neutralization (Fig. 2D).

### Engineered ACE2 broadly neutralizes SARS-CoV-2 variants, including Omicron, and other sarbecoviruses.

**Fig. 3.**
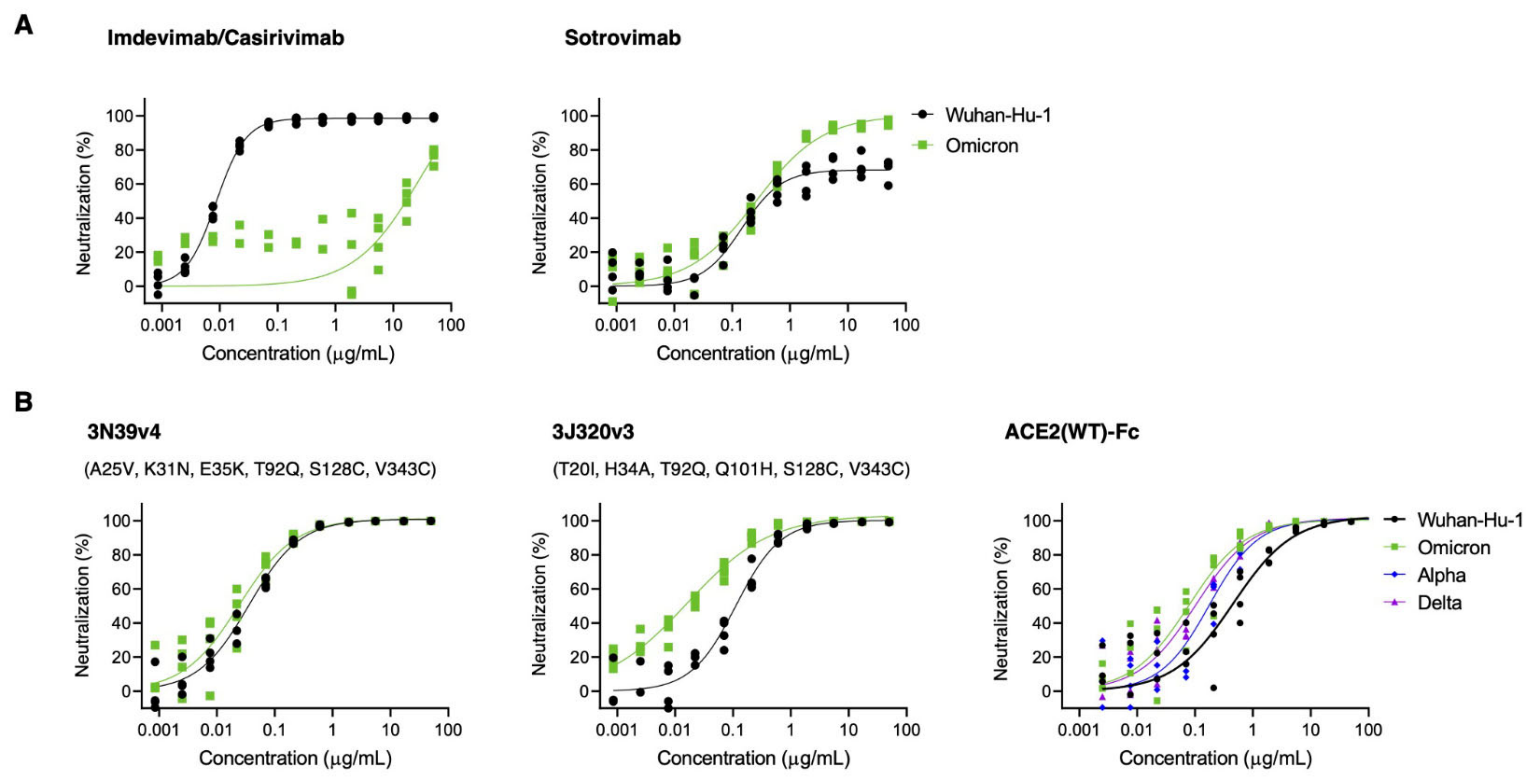
ACE2 decoys have intact neutralization efficacy against the Omicron variant. (**A**) Neutralization efficacy in 293T/ACE2 cells is shown for a cocktail of imdevimab and casirivimab (left) and monotherapy of sotrovimab (right)against Omicron pseudovirus. (**B**) Neutralization efficacy in 293T/ACE2 cells is shown for the 3N93v4 engineered ACE2 decoy (left), the 3J320v2 engineered ACE2 decoy (middle), or the wild type ACE2 decoy (right). The 3N39v4 and 3J320v3 have specific mutations providing high affinity toward the RBD and common S128C/V343C substitutions to inhibit ACE2 enzymatic activity. All adopted ACE2s were 740 amino acids with the collectrin domain. n = 4 technical replicates. The indicated concentrations of imdevimab and casirivimab were applied in a 1:1 ratio.

We next evaluated the efficacy of neutralizing therapeutics developed for the original Wuhan strain. Currently a cocktail of imdevimab and casirivimab and a monotherapy of sotrovimab are approved in Japan. The cocktail of imdevimab and casirivimab showed reduced neutralization activity against the Omicron variant (greater than a 1,000-fold reduction from Wuhan), whereas that of sotrovimab was preserved (Fig. 3A). We previously reported that engineered ACE2 could neutralize a broad range of SARS-CoV-2 variants including Alpha, Beta, and Gamma (*5*). The primary ACE2 mutant in the previous study, 3N39, carried seven mutations (A25V, K26E, K31N, E35K, N64I, L79F, and N90H). This mutant was further modified in later studies to reduce potential immunogenicity, motivating us to remove K26E, N64I, and L79F that made no or little contribution to the enhanced spike protein binding, and changed the glycan-eliminating N90H to T92Q, which we reasoned would have a similar effect. Furthermore, the ACE2 collectrin domain (residues 615 to 740) was appended to the mutant together with the introduction of a S128C/V343C disulfide to increase the production yield as well as to erase the enzyme activity. This resulted in what we call the 3N39v4 mutant. In the same fashion, 3J320v3 contains the collectrin domain and S128C/V343C. Despite far more extensive mutation in the Omicron RBD compared with previous VOCs, all three high-affinity engineered ACE2-Fc we developed (3N39v4, 3J113v2, and 3J320v3) exhibited high neutralizing efficacy against Omicron at concentrations indistinguishable or even higher than the original Wuhan strain (Fig. 3B and fig. S2). The concept of engineered ACE2 decoys has been independently introduced by several groups and their combination of mutations were all unique (*7*, *8*). Among these, we tested two different ACE2 mutants reported by Chan *et al*. (*7*), and both showed similar or better neutralization against Omicron (fig. S2). Wild type ACE2-Fc (spanning residues 18-740 with the collectrin domain) neutralized Omicron better than Wuhan, similar to the Alpha or Delta variants, which is consistent with a previous report (*9*). Nevertheless, engineered ACE2 molecules maintain an advantage for Omicron over wild type ACE2 decoy (Fig. 3B).

**Fig. 4.**
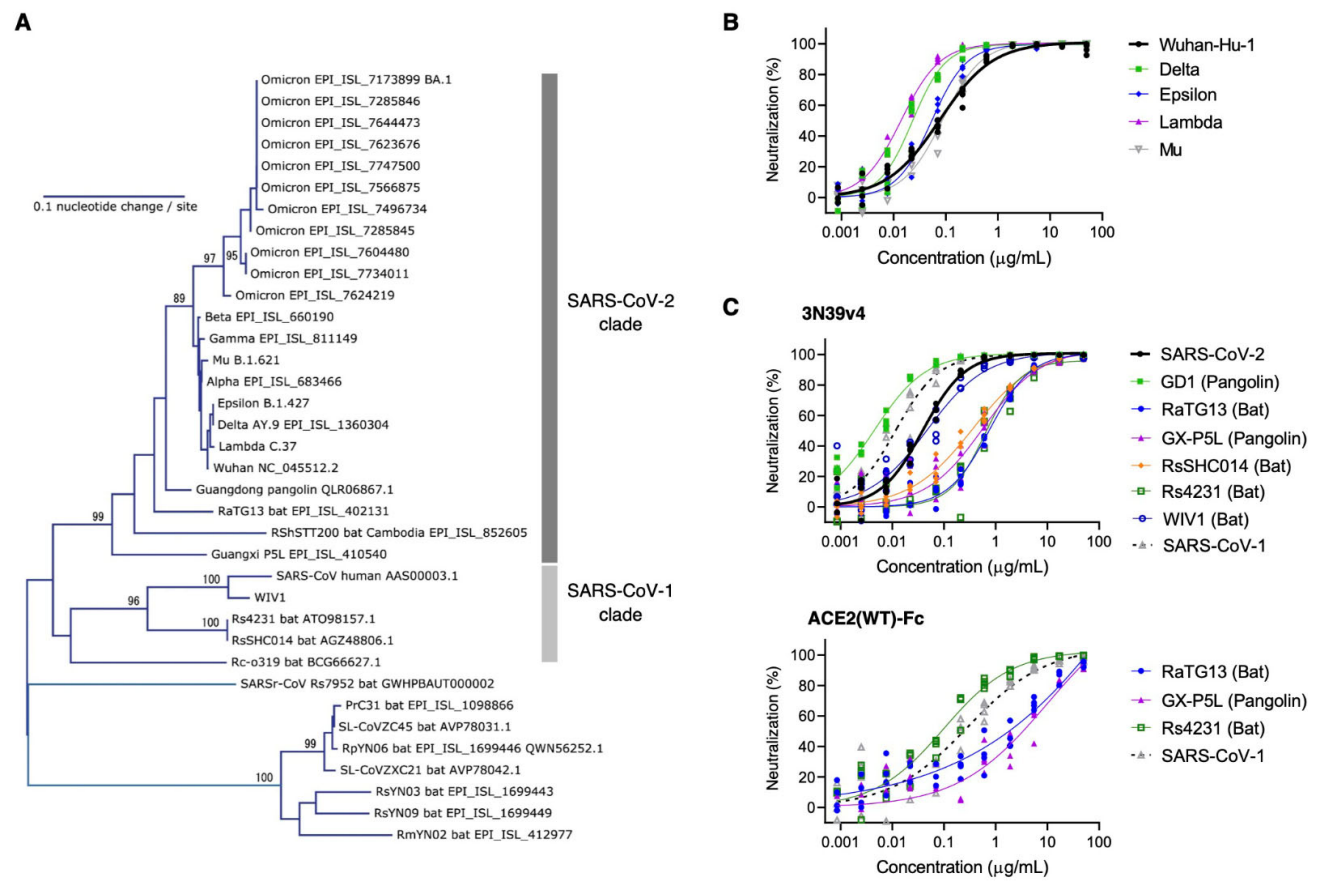
Neutralization assay for engineered ACE2 with pseudoviruses expressing spike proteins from SARS-CoV-2 variants and additional sarbecoviruses. (**A**) A phylogenetic tree of the RBD from variants of SARS-CoV-2 and relatives is shown. Accession number or lineage name is shown for each sequence. Bootstrap values larger than 80% are shown at the corresponding branches. (**B**) Neutralization efficacy was measured for engineered ACE2 (3N39v4) against previous SARS-CoV-2 variants in 293T/ACE2 cells. (**C**) Neutralization efficacy was measured for engineered ACE2(3N39v4) (top) or wild type ACE2(WT)-Fc (bottom) decoys against sarbecoviruses in 293T/ACE2 cells. n = 4 technical replicates.

We then examined the breadth of cross-neutralization against other SARS-CoV-2 variants and sarbecoviruses by our engineered ACE2 (Fig. 4A and fig. S3). Previously, we reported the effective neutralization against Alpha, Beta, and Gamma variants as well as SARS-CoV-1 (*5*). Here, engineered ACE2 (3N39v4) showed similar or better neutralization efficacy to the Delta, Epsilon, Lambda, or Mu variants as compared with the original Wuhan strain (Fig. 4B). Based on the broad representations of the RBD phylogenetic spectrum, 3 different viruses from the SARS-CoV-2 clade and 3 from the SARS-CoV-1 clade were also analyzed (*10*) (Fig. 4A and fig. S3). GD-1, RsSHC014, and WIV1 were tested as pseudoviruses harboring their own spike proteins, whereas RaTg13, GX-P5L, and Rs4231 were evaluated as chimeric spike proteins where their RBDs were inserted in the SARS-CoV-1 RBD region in order to obtain the enough infectivity for the analysis (*11*). ACE2 (3N39v4) inhibited GD-1, WIV1, and SARS-CoV-1 infection, although RaTg13, GX-P5L, and RsSHC014 were less neutralized by both engineered ACE2 and the wild type ACE2 decoy. Rs4231 was also less sensitive to engineered ACE2, but inhibited well by the wild type decoy (Fig. 4C). These results indicate that engineered ACE2 has therapeutic potency against a broad range of SARS-CoV-2 variants and some other sarbecoviruses.

### Engineered ACE2 confers protection against infection with authentic Omicron virus.

**Fig. 5.**
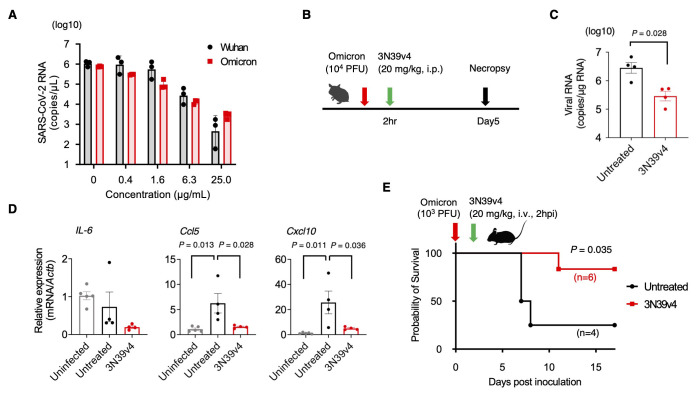
Engineered ACE2 neutralizes authentic Omicron and confers protection in hamsters and hACE2 transgenic mice. (**A**) Sensitivity of engineered ACE2 to the Wuhan and Omicron strains was compared by using each infectious virus in Vero E6/TMPRSS2 cells. RNA copy number was analyzed by qRT-PCR against nucleocapsid. n = 3 technical replicates. (**B**) Experimental scheme for the hamster model of SARS-CoV-2. (**C**) Quantification of viral RNA in the lungs of treated or untreated hamsters at day 5 post infection with the SARS-CoV-2 Omicron strain was performed by qRT-PCR against nucleocapsid. (n=4 per group). (**D**) Gene expression of inflammatory cytokines and chemokines was quantified by qRT-PCR in the hamsters. The expression of the gene encoding β-actin was used for normalization. (n=4). (**E**) A survival curve is shown for CAG-hACE2 Tg mice infected with the Omicron strain with or without 3N39v4 ACE2 decoy treatment (control: 2 male, 2 female, treated: 3 male and 3 female). *P*-values were determined by Mann-Whitney *U* test (C), one-way ANOVA and Tukey's multiple comparison test (D) and log-rank test (E).

Next, we examined the effects of our engineered ACE2 on propagation of authentic Omicron in vitro and in vivo. Vero E6 cells expressing transmembrane protease serine 2 (TMPRSS2) were infected with Wuhan or Omicron in the presence of engineered ACE2. Consistent with our pseudovirus data, sensitivity of SARS-CoV-2 Wuhan and Omicron variants to engineered ACE2 were comparable (Fig. 5A). The therapeutic potential of engineered ACE2 in animal models has been reported for both the parental Wuhan strain and for variants (*5*, *12*). We thus tested the therapeutic benefit of engineered ACE2 against Omicron as well. We infected Syrian hamsters by the intranasal route with 1 × 10^4^ plaque-forming units (PFU) of Omicron and then treated them with 20 mg/kg engineered ACE2 by intraperitoneal route 2 hours post-inoculation (Fig. 5B). After 5 days, viral RNA in the lungs was significantly suppressed by treatment with engineered ACE2 (p=0.028, Fig. 5C). Gene expression of inflammatory cytokines and chemokines showed reduction in *CCL5* and *CXCL10* transcription by treatment with engineered ACE2 (Fig. 5D). To confirm efficacy in a more severe model, we examined the effect of engineered ACE2 on survival of CAG-hACE2 mice that overexpress human ACE2 highly and ubiquitously and exhibit more severe SARS-CoV-2 transmission to the brain (*13*), as compared with K18-hACE2 mice directing epithelia-specific expression (*14*–*16*). The mice were challenged with 1 × 10^3^ PFU of Omicron through the intranasal route and engineered ACE2 was intravenously administered 2 hours post-inoculation at a dose of 20 mg/kg. Three out of 4 CAG-hACE2 mice died within 8 days after inoculation. In contrast, engineered ACE2 treatment significantly rescued these mice (p=0.035, Fig. 5E). These data indicate that engineered ACE2 remains active in neutralizing the Omicron variant in vitro and in vivo.

### Engineered ACE2 adjusts the side chain conformation of the mutated residues to bind to Omicron RBD.

The structure of the Omicron RBD complexed with wild type ACE2 has recently been determined by several groups (fig. S4A), revealing sustained binding affinity of this variant as compared to the original Wuhan strain by combination of both affinity-enhancing and reducing mutations (*17*–*19*). We previously determined the crystal structure of Wuhan RBD complexed with the original ACE2 mutant 3N39 (*5*). The wild type residue E35 forms an intramolecular salt bridge with K31 (fig.S4B) whereas the mutated K35 forms a direct hydrogen bond with Q493 of the Wuhan RBD in our structure (fig. S4C), leading us to speculate that the simultaneous mutations of E35K and K31N played a key role in the affinity enhancement of this mutant. On the other hand, Q493 was substituted with arginine in the Omicron RBD, allowing it to make a salt bridge with E35 of the wild type ACE2 (fig.S4D). We suspected that this could lower the affinity toward our engineered ACE2 due to electrostatic repulsion with K35. To simulate the binding mode, we built a homology model of a complex between the Omicron RBD (7t9l) and the ACE2 mutant 3N39 (7dmu). In the complex model, the electrostatic clash between K35 of the ACE2 mutant and R493 of Omicron RBD could easily be avoided by side chain rotation. Furthermore, we found that the side chain of R493 could form a direct hydrogen bond with N31 of ACE2(3N39) instead of K35 (fig. S4E). Therefore, consistent with the results of the neutralization assay, engineered ACE2 is expected to have no or little loss of affinity for the RBD of the Omicron variant.

### No complete escape mutation to engineered ACE2 was observed using deep mutational scanning of the RBD.

**Fig. 6.**
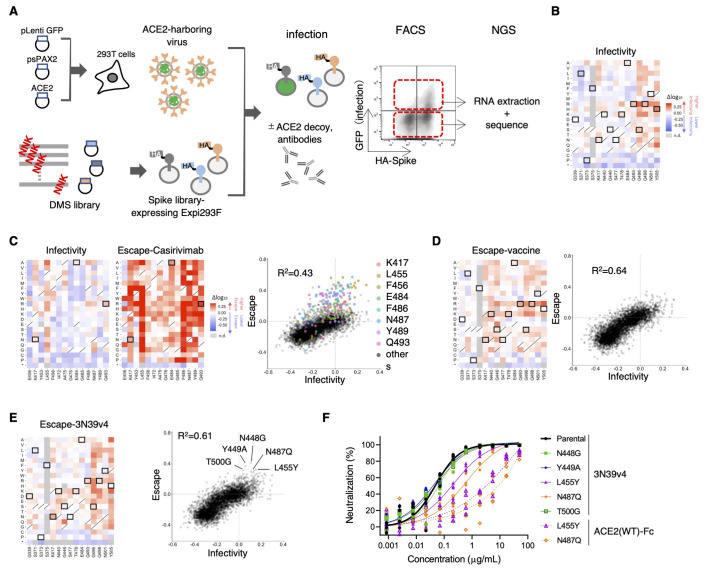
Deep mutational scanning identified no single-residue mutation to induce complete escape from engineered ACE2. (**A**) A schematic of the deep mutational scanning (DMS) approach to evaluate infectivity and escape from neutralizing agents is shown. FACS, fluorescence activated cell sorting. NGS, next generation sequencing. (**B**) Heatmaps illustrating how all single mutations that Omicron obtains affect its infectivity. Boxed squares indicate amino acid present in Omicron. Squares with a diagonal line through them indicate the original Wuhan strain amino acid. Heatmap squares are colored by mutational effect according to scale bars on the right. This and all subsequent coloring matches fig. S7 and S8. (**C**) The heatmaps show the alteration of infectivity and escape value from casirivimab in casirivimab antigen-binding sites of the spike protein. Boxed squares are mutations found in Omicron (left). Correlation in mutation effects on infectivity and escape from casirivimab are shown on the right. Colored dots are all amino acid substitution at the indicated major casirivimab antigen-binding sites. (**D**) The heatmap illustrates how all single mutations that Omicron obtained affect its escape from vaccinated serum samples. Boxed squares indicate amino acid present in Omicron (left). Correlations in mutation effects on infectivity and escape from vaccinated serum are shown on the right. (**E**) The heatmap illustrates how all single mutations that Omicron obtained affect its escape from 3N39v4. Boxed squares indicate amino acid present in Omicron (left). The dot plot shows the correlation between Omicron infectivity and escape from 3N39v4. Indicated mutants were individually analyzed in (F). (**F**) Neutralization efficacy was measured in 293T/ACE2 cells for 3N39v4 or wild type ACE2 decoys against the five indicated escape candidates. n = 4 technical replicates.

To comprehensively analyze the alteration of viral infectivity and escape from neutralizing agents, we performed a deep mutational scan (DMS) of the RBD in the context of full-length spike protein expressed on human Expi293F cells (*20*), followed by incubation with ACE2-harboring green fluorescent protein (GFP)-reporter viruses. In this “inverted orientation” setting, ACE2-expressing virions efficiently infected spike protein-expressing cells only (fig. S5A). Although DMS analysis on the spike protein had been done by Starr *et al*. (*21*), this prior study used yeast surface display of isolated RBDs, which would assess the RBD’s structural stability and its affinity against ACE2 or neutralizing agents separately. In contrast, Chan *et al*. used Expi293F cells expressing full-length spike protein to evaluate the affinity for wild type and engineered ACE2 (*20*). We modified their screening system to monitor the fusion of cell and viral membranes mediated by interactions between spike protein and ACE2, which better mimics the true infection process, albeit in an inverted orientation. The hemagglutinin (HA)-tagged spike protein library encompassed all 20 amino acid substitutions in the RBD (encompassing P329 to G538) of the original SARS-CoV-2 Wuhan strain spike protein and was transfected in Expi293F cells in a manner where cells typically acquire no more than a single variant (*20*). After infection of library cells by ACE2-harboring viruses in the presence or absence of neutralizing agents, infected GFP-positive cells and control GFP-negative cells were harvested with fluorescence-activated cell sorting (FACS), RNA was extracted, and the library was sequenced (Fig. 6A). The resulting spike protein-expressing cells constituted approximately 15% of transfected cells, which is a reasonable rate to avoid multiple library induction (*22*). For infectivity analysis, ACE2-harboring viruses were titrated to induce infection in 2 to 3% of the cells in consideration of the detection range and library complexity (fig. S5B). To analyze escape ability, ACE2-harboring viruses and neutralization agents were optimized to observe the reduction of infectivity from 10% to 3% (fig. S5B and C). DMS experiments were performed in duplicate, which produced similar results (R^2^ = ~0.5) as was the case in previous studies conducted on HIV and influenza pathology with library viruses (*23*, *24*) (fig. S6A). We also performed DMS for spike protein expression assessed by the staining of HA tagged to N-terminal full-length spike protein. Among about 15% of HA-positive cells, the top 25% and bottom 25% of cells were sorted (fig. S6B). The resulting single mutant count frequencies correlated well between independent duplicate experiments (R^2^ = 0.93; fig. S6C). The scan without neutralizing agents provided information about infectivity alteration due to all single amino acid mutations in the RBD (fig. S7A). When our DMS data for infectivity and expression were compared with those obtained in the previous yeast surface display DMS from Starr *et al*. (*21*), infectivity was weakly correlated with the prior study’s affinity data, which was based on ACE2-binding at several ACE2 concentrations (R^2^ = 0.23). In contrast, expression data correlated better, in spite of the difference in using full-length spike protein versus isolated RBD (R^2^ = 0.48; fig. S7B to E).

According to the scanning data for infectivity, 8 of 15 mutations in the RBD of Omicron may enhance infectivity, whereas 3 mutations (S371L, S373P, S375F) that exist in the conserved region may reduce infection (Fig. 6B). Incubation of library cells and ACE2-harboring viruses with neutralizing drug and serum succeeded in revealing the mutational patterns that enable evasion. For example, the scan in the presence of casirivimab detected all epitope residues previously reported (*25*, *26*), which confirmed that this assay had adequate sensitivity to detect actual escape mutations (Fig. 6C and fig. S8A). In this panel, Omicron mutation, K417N, E484A and Q493R were all found to contribute to escape from casirivimab (Fig. 6C). Some VOCs have exhibited partial resistance to neutralization by serum from immunized individuals (*27*); however, actual escape is achieved by a combination of mutations, not by any single mutation (*28*). Consistently, the scan measuring resistance to serum samples from vaccinated individuals revealed that alteration of escape value simply paralleled that of infectivity without irregular enhancements, unlike the case of casirivimab (Fig. 6D, fig. S8B). A similar result was also observed in a scan for escape from engineered ACE2 (Fig. 6E and fig. S8C). The close correlation between escape value and infectivity was reproduced by serum from a different vaccinated individual (fig. S9). When major escape mutants obtained through the DMS were individually subjected to the neutralization assay, it was found that L455Y and N487Q partially reduced the neutralization efficacy of both 3N39v4 and wild type ACE2 decoys (Fig. 6F). However, engineered ACE2 still maintained a neutralization efficacy similar to the degree of sotrovimab for both the parental Wuhan strain and the Omicron variant (Fig. 3A). These results demonstrate that engineered ACE2 effectively neutralized each single-residue mutation-expressing virus. Finally, we confirmed that both 3N39v4 and 3J320v3 retained neutralization efficacy against the Omicron subvariant BA.2 (fig. S10).

## DISCUSSION

The Omicron variant contains approximately 30 mutations in the spike protein and escapes vaccine, convalescent serum, and some therapeutic antibodies (*1*–*4*, *29*–*32*). Intensive analyses from all over the world indicate a 10 to 50-fold reduction in neutralization titers for Omicron in vaccines and convalescent individuals infected with different variants (*29*, *30*, *32*). Consistently, our assay using Omicron pseudovirus revealed that 17.7-fold and 9.5 to 19.3-fold reduction in serum samples from individuals vaccinated with two doses of BNT162b2-vaccinated and previously infected people, respectively. The assay using pseudovirus harboring chimeric spike protein of Omicron and the original Wuhan strain demonstrated that multiple mutations in the NTD could contribute to reduced neutralization in addition to escape due to RBD mutations. A previous study showed that a deletion mutation in the NTD resulted in viral escape from isolated NTD-binding neutralizing antibodies (*33*). According to another report, vaccinated and convalescent serum samples exhibited weak neutralization titers, even after depletion of RBD-binding antibodies (*34*). Furthermore, direct structural analysis of NTD-targeted neutralizing antibodies indicated that they each recognize a common glycan-free, electropositive surface comprised of flexible loops where most Omicron NTD mutations are located (*6*). These reports point to the possibility of NTD-mediated active neutralization by serum from immunized individuals. Consistent with this idea, the present study demonstrates that the extensive escape of Omicron can be achieved, in part, by attenuated neutralization of NTD-binding antibodies. Regarding the escape due to RBD mutations, serum samples from immunized individuals contain polyclonal antibodies and are less affected by single mutations. However, a previous DMS study identified RBD positions F456 and E484 mutated to alanine in Omicron, as major epitopes recognized by serum from immunized individuals (*34*).

For therapeutic strategies to prevent mutational escape, some antibodies are used in the form of a cocktail and others are designed to target a conserved region. The present study showed that a cocktail of imdevimab and casirivimab failed to neutralize Omicron, whereas sotrovimab, which targets a highly conserved epitope, remained effective. Other papers also reported impaired neutralization in a wide range of monoclonal antibodies (*2*–*4*, *31*), and that only sotrovimab was minimally affected among those in clinical use (*2*). Omicron exhibits the ability to evade combinations of antibodies and has acquired mutations even in highly conserved sites (S371L, S373P, S375F). These observations provide a sobering outlook for the therapeutic development of monoclonal antibodies against viruses in the context of escape mutations. In contrast, engineered ACE2 successfully neutralized Omicron and other sarbecoviruses. The emergence of the Omicron variant reinforces the difficulty of drug development against viral infection and highlights the strength of receptor decoys as a therapeutic strategy.

In this study, we performed DMS using full-length spike protein-expressing human cells and an ACE2-harboring virus. Incubation of these components aimed to functionally reproduce infection of host cells. In contrast, another study performed DMS with yeast surface display and analyzed RBD expression and binding of RBD with several concentrations of ACE2 (*21*). Yeast screening has advantages in terms of library size and the ability to restrict incorporation of multiple mutants due to the exclusive nature of different plasmids. Mammalian cell-based screening has a limit in library size and can be contaminated by cells expressing multiple mutants; however, its strength is that it allows analysis of various phenotypes, including virus infection. When compared with the yeast surface display DMS, the pattern of effects on protein expression showed similar trends, in spite of difference in the form of spike protein used. On the other hand, our infectivity DMS was less correlated with ACE2 affinity data collected from the yeast surface display DMS study. One explanation for this difference was the setting of assay. Our DMS reflected the binding and subsequent fusion of virus and cell membrane, whereas the yeast surface display DMS analyzed the absolute amount of bound soluble ACE2 in serial concentrations. However, intensive validation of each DMS system is required for further discussion.

Through DMS, we found that L455Y and N487Q mutations mildly reduced neutralization efficacy of engineered ACE2. These residues make direct contacts with ACE2, but are separate from the affinity-enhancing mutations of engineered ACE2 (*5*). As a result, these mutations also caused a reduction in the neutralization ability of wild type ACE2. In the case of N487Q, both DMS results showed elevated infectivity, increased expression, and reduced ACE2 binding affinity. It is thus possible that reduced ACE2 affinity is offset by increased spike protein expression and stability to maintain overall infectivity, while causing attenuation of the neutralization ability of ACE2 decoys.

Another study also reported that certain RBD mutations affected other high-affinity ACE2 decoys (*35*). In theory, similar viral mutations that reduce the neutralization efficacy of engineered ACE2 may arise. However, the virus would not encounter such agents unless they were used as a therapeutic intervention. In contrast, viruses circulating in the wild are under selection pressure to escape neutralizing antibodies due to vaccination, prior infection, or both. Therefore, preferential emergence of ACE2 escape mutants is potentially less likely.

Our study has several limitations. First, the engineered ACE2 is not perfect at neutralizing all sarbecoviruses. Among the seven representative sarbecoviruses that were analyzed to demonstrate the broad efficacy of engineered ACE2, the viruses phylogenetically closer to SARS-CoV-2 and SARS-CoV-1 were more sensitive to engineered ACE2. It is clear that more extensive analysis is required to accurately predict the range of efficacy of each engineered ACE2. Second, we noted that several spike clones with apparent stop codons exhibited near-neutral infectivity and immune escape in our DMS experiments, which suggests that the transfections had delivered multiple library members into the same cell, despite our experimental design to avoid such co-transfection. Lastly, the DMS did reveal a few single amino acid substitutions that reduced sensitivity to neutralization by engineered ACE2. Although the engineered ACE2 maintained reasonable neutralizing activity toward each of these mutants, it will be imperative to examine the effect of their combination. Despite these limitations, our results suggest that an engineered ACE2 decoy is a robust therapeutic modality with utility against broad range of SARS-CoV-2 variants and sarbecoviruses. It is also promising in the context of efforts to stockpile medical assets as a countermeasure against future zoonotic coronavirus diseases.

## MATERIALS AND METHODS

### Study Design

Research blood samples were obtained from hospitalized adults with polymerase chain reaction (PCR)-confirmed SARS-CoV-2 infection and vaccinated individuals who were enrolled in a prospective cohort study approved by the Clinical Research Review Committee in Kyoto Prefectural University of medicine (ERB-C-1954-1). All human participants provided written informed consent. We collected peripheral blood from 12 individuals (7 males, 5 females, age 24 to 56, mean 37.5) at 3 months post vaccination with two doses of the Pfizer-BioNTech vaccine, BNT162b2. Peripheral blood was also collected from 11 SARS-CoV-2 convalescent individuals (7 males, 4 females, age 50 to 83, mean 69) before the Delta variant wave (December 2020 through January 2021), and 18 SARS-CoV-2 convalescent individuals (15 males, 3 females, age 36 to 77, mean 56.6) during or after the Delta wave (August 2021 through October 2021). The study size was determined by the number of samples that were available from the cohort study and not based on any power calculations. Experiments described in this manuscript were not performed blinded.

All animal experiments with SARS-CoV-2 were performed in biosafety level 3 (ABSL3) facilities at the Research Institute for Microbial Diseases, Osaka University. The study protocol was approved by the Institutional Committee of Laboratory Animal Experimentation of the Research Institute for Microbial Diseases, Osaka University (R02-08-0). All efforts were made during the study to minimize animal suffering and to reduce the number of animals used in the experiments.

### Omicron genetic and epidemic analysis

Amino acid mutation frequencies in the spike protein for each variant (Alpha = 1,138,704, Beta = 40,135, Gamma = 117,200, Delta = 3,441,137, and Omicron = 5,469 sequences) were extracted from the report of outbreak.info (https://outbreak.info/compare-lineages?pango=Delta&pango=Omicron&pango=Alpha&pango=Beta&pango=Gamma&gene=S&threshold=0.2&nthresh=1&sub=false&dark=true%29.%20Accessed%2017%20December%202021.) as of December 17, 2021. Mutation frequencies of each amino acid substitution which was observed in Omicron were plotted using “corrplot” package in R. Time course of variant distribution was analyzed by Nextclade ver 1.7.0 (https://joss.theoj.org/papers/10.21105/joss.03773) from SARS-CoV-2 nucleic acid sequences which were downloaded from GISAID database as of December 17, 2021. A phylogenetic tree of spike proteins from SARS-CoV-2 variants and relatives were inferred based on full-length amino acid sequences taken from GISAID, GenBank, National Genomics Data Center, and Coronavirus Antiviral & Resistance Database of Stanford University (https://covdb.stanford.edu/). The neighbor joining method (*36*) was applied to a distance matrix estimated by the maximum likelihood method. To perform multiple sequence alignment (MSA) of spike proteins, 6,000,693 spike protein amino acid sequences were downloaded from GISAID on Dec 14, 2021. The frequency of all unique RBD regions (defined as R319-541F) were determined for each major variant (as defined in https://www.who.int/en/activities/tracking-SARS-CoV-2-variants/), and the most frequent instance within a variant was used as a representative. The sequences were multiply aligned by Multiple alignment by fast Fourier transform (MAFFT) (*37*) with the corresponding RBD regions from PG-GD1, VIW1 and CoV-1. The MSA was rendered using an in-house script to show only positions non-identical to the Wuhan strain. The code is available at Zenodo (DOI: 10.5281/zenodo.6440010).

### Cell culture

Lenti-X 293T cells (Clontech) and its derivative, 293T/ACE2 cells were cultured at 37 °C with 5% CO_2_ in Dulbecco’s modified Eagle’s medium (DMEM, WAKO) containing 10% fetal bovine serum (Gibco) and penicillin/streptomycin (100 U/ml, Invitrogen). Vero E6/TMPRSS2 cells were a gift from National Institutes of Biomedical Innovation, Health and Nutrition (Japan) and cultured at 37°C with 5% CO2 in DMEM (WAKO) containing 5% fetal bovine serum (Gibco) and penicillin/streptomycin (100 U/ml, Invitrogen). All cell lines routinely tested negative for mycoplasma contamination.

### Protein synthesis and purification

Monoclonal antibodies and engineered ACE2 were expressed using the Expi293F cell expression system (Thermo Fisher Scientific) according to the manufacturer’s protocol. Fc-fusion proteins were purified from conditioned media using the rProtein A Sepharose Fast Flow (Cytiva). Fractions containing target proteins were pooled and dialyzed against phosphate buffered saline (PBS).

### Pseudotyped virus neutralization assay

Pseudotyped reporter virus assays were conducted as previously described (*5*). With a plasmid encoding the SARS-CoV-2 spike protein (addgene #145032) as a template, mutations for variants of concern, including omicron BA.1 and BA.2, as well as the ΔC19 deletion (with 19 amino acids deleted from the C terminus) were cloned into pcDNA4TO (Invitrogen) (*38*). Pangolin CoV GD-1, Bat CoV RsSHC014, and WIV1 spike proteins were synthesized (Integrated DNA Technologies) and cloned into pcDNA4TO (Invitrogen) in the form of ΔC19 (*10*, *39*). Pangolin CoV GX-P5L, Bat CoV RaTG13, and Rs4231 RBDs were synthesized (Integrated DNA Technologies) and cloned into the RBD of SARS-CoV-1 (ΔC19) (*5*, *10*, *11*, *39*). Spike protein-expressing pseudoviruses with a luciferase reporter gene was prepared by transfecting plasmids (OmicronΔC19, psPAX2-IN/HiBiT (*40*), and pLenti firefly) into LentiX-293T cells with Lipofectamine 3000 (Invitrogen). After 48 hours, supernatants were harvested, filtered with a 0.45 μm low protein-binding filter (SFCA), and frozen at –80 °C. The 293T/ACE2 cells were seeded at 10,000 cells per well in 96-well plates. HiBit value-matched pseudoviruses and three-fold dilution series of serum or therapeutic agents were incubated for 1 hour, then this mixture was added to 293T/ACE2 cells. After a 1 hour pre-incubation, medium was changed. At 48 hours post infection, cellular expression of the luciferase reporter, indicating viral infection, was determined using ONE-Glo Luciferase Assay System (Promega). Luminescence was read on Infinite F200 pro system (Tecan). The assay for each serum sample was performed in triplicate, and the 50% neutralization titer was calculated using Prism version 9 (GraphPad Software).

### Library construction, FACS, and Illumina sequencing analysis

Saturation mutagenesis was focused on the original Wuhan strain spike residues F329 to C538, forming the RBD. Pooled oligos with degenerate NNK codons were synthesized by Integrated DNA Technologies, Inc. Synthesized oligos were extended by overlap PCR and cloned into pcDNA4TO HMM38-HA-full length spike plasmids. Transient transfection conditions were used that typically provide no more than a single coding variant per cell (*20*). Expi293F cells at 2 × 10^6^ cells per ml were transfected with a mixture of 1 ng of library plasmid with 1 μg of pMSCV as a junk plasmid per ml using ExpiFectamine (Thermo Fisher Scientific). Twenty-four hours after transfection, cells were incubated with ACE2-harboring green fluorescent protein (GFP) reporter viruses, which were generated by transfecting pcDNA4TO ACE2, psPAX2 (addgene #12260), and pLenti GFP into LentiX-293T cells with Lipofectamine 3000 (Invitrogen). The viruses have ACE2 on the surface instead of glycoprotein and can infect the spike protein-expressing cells. In case of escape analysis, cells were pre-incubated with neutralizing agents for 1 hour. After the 1 hour incubation with ACE2-harboring virus, the medium was replaced and cells were collected 24 hours after infection for FACS. Cells were washed twice with PBS containing 10% bovine serum albumin (BSA) and then co-stained for 20 min with anti-hemagglutinin (HA) Alexa Fluor 647 (clone TANA2,1:4000 dilution; MBL). Cells were again washed twice before sorting on a MA900 cell sorter (Sony). Dead cells, doublets, and debris were excluded by first gating on the main population by forward and side scatter. From the HA positive (Alexa Fluor 647 positive) population, GFP-positive and -negative cells were collected. The total numbers of collected cells were about 2 million cells for each group. Total RNA was extracted from collected cells TRIzol (Life Technologies) and Direct-zol RNA MiniPrep (Zymo Research Corporation) according to the manufacturer's protocol. First-strand complementary DNA (cDNA) was synthesized with PrimeScript II Reverse Transcriptase (Takara) primed with a gene-specific oligonucleotide. Libraries were designed for 3 sections separately in the RBD and then pooled. After cDNA synthesis, each library was amplified with specific primers. Following a second round of PCR, primers added adapters for annealing to the Illumina flow cell and sequencing primers, together with barcodes for experiment identification. The PCR products were sequenced on an Illumina NovaSeq 6000 using a 2 × 150 nucleotide paired-end protocol in Department of Infection Metagenomics, Research Institute for Microbial Diseases, Osaka University. Data were analyzed comparing the read counts with each group normalized relative to the wild type sequence read-count. Log10 enrichment ratios for all the individual mutations were calculated and normalized by subtracting the log10 enrichment ratio for the wild type sequence across the same PCR-amplified fragment.

### Viruses

The SARS-CoV-2 (Wuhan: 2019-nCoV/Japan/TY/WK-521/2020, Omicron: 2019-nCoV/Japan/TY38-873/2021) strain was isolated at National Institute of Infectious Diseases (NIID). SARS-CoV-2 viruses were propagated in Vero E6/TMPRSS2 cells. Viral supernatant was harvested at two days post-infection and viral titers were determined by plaque assay.

### SARS-CoV-2 neutralization assay

Vero E6/TMPRSS2 cells were seeded at 80,000 cells in 24 well plates and incubated overnight. Then, SARS-CoV-2 was infected at multiplicity of infection of 0.1 together with soluble ACE2-Fc protein. After 2 hours, cells were washed with fresh medium and incubated with fresh medium for 22 hours. Culture supernatants were collected and analyzed by quantitative real-time reverse transcriptase-polymerase chain reaction (qRT-PCR) assay.

### Animal models of SARS-CoV-2 infection

Four week-old male Syrian hamsters were purchased from SLC Japan. Hamsters were anaesthetized by intraperitoneal administration of 0.75 mg/kg medetomidine (Meiji Seika), 2 mg/kg- midazolam (Sandoz) and 2.5 mg/kg butorphanol tartrate (Meiji Seika) and challenged with 1.0 × 10^4^ PFU of SARS-CoV-2 Omicron (in 60μL) by the intranasal route (30μL per nostril). After 2 hours post inoculation, ACE2-Fc (3N39v2, 20mg/kg) were administered by intraperitoneal injection. On day 5 post inoculation, all animals were euthanized and lungs were collected to analyze the virus genome copy number and host gene expression by qRT-PCR.

CAG-hACE2 transgenic mice (hACE2-Tg) were obtained from the Laboratory Animal Resource Bank of the National Institutes of Biomedical Innovation, Health and Nutrition (NIBIOHN). These mice were maintained by crossing with wild type C57BL/6 mice. The primers for genotyping were 5′-CTTGGTGATATGTGGGGTAGA-3′ and 5′-CGCTTCATCTCCCACCACTT-3′ as shown previously (*13*). Male and female mice aged at four-week-old were anaesthetized and challenged with 1.0 × 10^3^ PFU of SARS-CoV-2 Omicron (in 20μL) by the intranasal route (10μL per nostril). At 2 hours post inoculation, ACE2-Fc (3N39v2, 20mg/kg) was administered by intravenous injection. Mice were monitored daily for survival.

### Quantitative RT-PCR

Total RNA of lung homogenates was isolated using ISOGENE II (NIPPON GENE). Real-time RT-PCR was performed with the Power SYBR Green RNA-to-CT 1-Step Kit (Applied Biosystems) using a AriaMx Real-Time PCR system (Agilent). The relative quantitation of target mRNA expression was performed using the 2-ΔΔCT method. The values were normalized by those of the housekeeping gene, β-actin. The following primers were used: for *Actb* (encoding β-actin), 5′-TTGCTGACAGGATGCAGAAG-3′ and 5′-GTACTTGCGCTCAGGAGGAG-3′; for 2019-nCoV_N2, 5′-AAATTTTGGGGACCAGGAAC-3′ and 5′-TGGCAGCTGTGTAGGTCAAC-3′; for *Il6*, 5′-GGACAATGACTATGTGTTGTTAGAA-3′ and 5′-AGGCAAATTTCCCAATTGTATCCAG-3′; for *Ccl5*, 5′-TCAGCTTGGTTTGGGAGCAA-3′ and 5′-TGAAGTGCTGGTTTCTTGGGT-3′; and for *Cxcl10*, 5′-TACGTCGGCCTATGGCTACT-3′and 5′-TTGGGGACTCTTGTCACTGG-3′.

### Quantitative RT-PCR of Viral RNA in the supernatant

The amount of RNA copies in the culture medium was determined using a qRT-PCR assay as previously described with slight modifications (*41*). In brief, 5 μl of culture supernatants were mixed with 5 μl of 2× RNA lysis buffer (2% Triton X-100, 50 mM KCl, 100 mM Tris-HCl [pH 7.4], 40% glycerol, 0.4 U/μl of Superase IN [Life Technologies]) and incubated at room temperature for 10 min, followed by addition of 90 μl of RNase free water. Next, 2.5 μl of volume of the diluted samples was added to 17.5 μl of the reaction mixture. Real-time RT-PCR was performed with the Power SYBR Green RNA-to-CT 1-Step Kit (Applied Biosystems) using a AriaMx Real-Time PCR system (Agilent).

### Statistical analysis

Neutralization measurements were done in technical triplicates and relative luciferase units were converted to percent neutralization and plotted with a non-linear regression model to determine 50% neutralization titer (NT_50_) values using GraphPad Prism software (version 9.0.0). Comparisons between two groups were made with paired *t* tests, Mann-Whitney *U* tests, and log-rank tests. More than two groups were compared by one-way ANOVA and Tukey’s multiple comparison tests.

## Supplementary Materials

### Other Supplementary Material for this manuscript includes the following:

MDAR Reproducibility Checklist

## References

[1] S. Collie , J. Champion , H. Moultrie , L. G. Bekker , G. Gray , Effectiveness of BNT162b2 Vaccine against Omicron Variant in South Africa. N. Engl. J. Med. 386, 494–496 (2022). 34965358 10.1056/NEJMc2119270 PMC8757569

[2] L. A. VanBlargan , J. M. Errico , P. J. Halfmann , S. J. Zost , J. E. Crowe Jr ., L. A. Purcell , Y. Kawaoka , D. Corti , D. H. Fremont , M. S. Diamond , An infectious SARS-CoV-2 B.1.1.529 Omicron virus escapes neutralization by therapeutic monoclonal antibodies. Nat. Med. 28, 490–495 (2022). 10.1038/s41591-021-01678-y 35046573 PMC8767531

[3] M. Hoffmann , N. Kruger , S. Schulz , A. Cossmann , C. Rocha , A. Kempf , I. Nehlmeier , L. Graichen , A. S. Moldenhauer , M. S. Winkler , M. Lier , A. Dopfer-Jablonka , H. M. Jack , G. M. N. Behrens , S. Pohlmann , The Omicron variant is highly resistant against antibody-mediated neutralization: Implications for control of the COVID-19 pandemic. Cell 185, 447–456.e11 (2022). 35026151 10.1016/j.cell.2021.12.032 PMC8702401

[4] D. Planas , N. Saunders , P. Maes , F. Guivel-Benhassine , C. Planchais , J. Buchrieser , W. H. Bolland , F. Porrot , I. Staropoli , F. Lemoine , H. Pere , D. Veyer , J. Puech , J. Rodary , G. Baele , S. Dellicour , J. Raymenants , S. Gorissen , C. Geenen , B. Vanmechelen , T. Wawina-Bokalanga , J. Marti-Carreras , L. Cuypers , A. Seve , L. Hocqueloux , T. Prazuck , F. Rey , E. Simon-Loriere , T. Bruel , H. Mouquet , E. Andre , O. Schwartz , Considerable escape of SARS-CoV-2 Omicron to antibody neutralization. Nature 602, 671–675 (2022). 35016199 10.1038/s41586-021-04389-z

[5] Y. Higuchi , T. Suzuki , T. Arimori , N. Ikemura , E. Mihara , Y. Kirita , E. Ohgitani , O. Mazda , D. Motooka , S. Nakamura , Y. Sakai , Y. Itoh , F. Sugihara , Y. Matsuura , S. Matoba , T. Okamoto , J. Takagi , A. Hoshino , Engineered ACE2 receptor therapy overcomes mutational escape of SARS-CoV-2. Nat. Commun. 12, 3802 (2021). 10.1038/s41467-021-24013-y 34155214 PMC8217473

[6] G. Cerutti , Y. Guo , T. Zhou , J. Gorman , M. Lee , M. Rapp , E. R. Reddem , J. Yu , F. Bahna , J. Bimela , Y. Huang , P. S. Katsamba , L. Liu , M. S. Nair , R. Rawi , A. S. Olia , P. Wang , B. Zhang , G. Y. Chuang , D. D. Ho , Z. Sheng , P. D. Kwong , L. Shapiro , Potent SARS-CoV-2 neutralizing antibodies directed against spike N-terminal domain target a single supersite. Cell Host Microbe 29, 819–833.e7 (2021). 10.1016/j.chom.2021.03.005 33789084 PMC7953435

[7] K. K. Chan , D. Dorosky , P. Sharma , S. A. Abbasi , J. M. Dye , D. M. Kranz , A. S. Herbert , E. Procko , Engineering human ACE2 to optimize binding to the spike protein of SARS coronavirus 2. Science 369, 1261–1265 (2020). 10.1126/science.abc0870 32753553 PMC7574912

[8] A. Glasgow , J. Glasgow , D. Limonta , P. Solomon , I. Lui , Y. Zhang , M. A. Nix , N. J. Rettko , S. Zha , R. Yamin , K. Kao , O. S. Rosenberg , J. V. Ravetch , A. P. Wiita , K. K. Leung , S. A. Lim , X. X. Zhou , T. C. Hobman , T. Kortemme , J. A. Wells , Engineered ACE2 receptor traps potently neutralize SARS-CoV-2. Proc. Natl. Acad. Sci. U.S.A. 117, 28046–28055 (2020). 10.1073/pnas.2016093117 33093202 PMC7668070

[9] K. M. Hastie , H. Li , D. Bedinger , S. L. Schendel , S. M. Dennison , K. Li , V. Rayaprolu , X. Yu , C. Mann , M. Zandonatti , R. Diaz Avalos , D. Zyla , T. Buck , S. Hui , K. Shaffer , C. Hariharan , J. Yin , E. Olmedillas , A. Enriquez , D. Parekh , M. Abraha , E. Feeney , G. Q. Horn , Y. Aldon , H. Ali , S. Aracic , R. R. Cobb , R. S. Federman , J. M. Fernandez , J. Glanville , R. Green , G. Grigoryan , A. G. Lujan Hernandez , D. D. Ho , K. A. Huang , J. Ingraham , W. Jiang , P. Kellam , C. Kim , M. Kim , H. M. Kim , C. Kong , S. J. Krebs , F. Lan , G. Lang , S. Lee , C. L. Leung , J. Liu , Y. Lu , A. MacCamy , A. T. McGuire , A. L. Palser , T. H. Rabbitts , Z. Rikhtegaran Tehrani , M. M. Sajadi , R. W. Sanders , A. K. Sato , L. Schweizer , J. Seo , B. Shen , J. L. Snitselaar , L. Stamatatos , Y. Tan , M. T. Tomic , M. J. van Gils , S. Youssef , J. Yu , T. Z. Yuan , Q. Zhang , B. Peters , G. D. Tomaras , T. Germann , E. O. Saphire ; CoVIC-DB team1 , Defining variant-resistant epitopes targeted by SARS-CoV-2 antibodies: A global consortium study. Science 374, 472–478 (2021). 10.1126/science.abh2315 34554826 PMC9302186

[10] C. W. Tan , W. N. Chia , B. E. Young , F. Zhu , B. L. Lim , W. R. Sia , T. L. Thein , M. I. Chen , Y. S. Leo , D. C. Lye , L. F. Wang , Pan-Sarbecovirus Neutralizing Antibodies in BNT162b2-Immunized SARS-CoV-1 Survivors. N. Engl. J. Med. 385, 1401–1406 (2021). 10.1056/NEJMoa2108453 34407341 PMC8422514

[11] M. Letko , A. Marzi , V. Munster , Functional assessment of cell entry and receptor usage for SARS-CoV-2 and other lineage B betacoronaviruses. Nat. Microbiol. 5, 562–569 (2020). 10.1038/s41564-020-0688-y 32094589 PMC7095430

[12] L. Zhang , S. Dutta , S. Xiong , M. Chan , K. K. Chan , T. M. Fan , K. L. Bailey , M. Lindeblad , L. M. Cooper , L. Rong , A. F. Gugliuzza , D. Shukla , E. Procko , J. Rehman , A. B. Malik , Engineered ACE2 decoy mitigates lung injury and death induced by SARS-CoV-2 variants. Nat. Chem. Biol. 18, 342–351 (2022). 10.1038/s41589-021-00965-6 35046611 PMC8885411

[13] M. N. Asaka , D. Utsumi , H. Kamada , S. Nagata , Y. Nakachi , T. Yamaguchi , Y. Kawaoka , K. Kuba , Y. Yasutomi , Highly susceptible SARS-CoV-2 model in CAG promoter-driven hACE2-transgenic mice. JCI Insight 6, e152529 (2021). 10.1172/jci.insight.152529 34463644 PMC8525594

[14] A. A. Dolskiy , A. S. Gudymo , O. S. Taranov , I. V. Grishchenko , E. M. Shitik , D. Y. Prokopov , V. O. Soldatov , E. V. Sobolevskaya , S. A. Bodnev , N. V. Danilchenko , A. A. Moiseeva , P. Y. Torzhkova , Y. A. Bulanovich , G. S. Onhonova , E. K. Ivleva , M. V. Kubekina , A. E. Belykh , T. V. Tregubchak , A. B. Ryzhikov , E. V. Gavrilova , R. A. Maksyutov , A. V. Deykin , D. V. Yudkin , The Tissue Distribution of SARS-CoV-2 in Transgenic Mice With Inducible Ubiquitous Expression of hACE2. Front. Mol. Biosci. 8, 821506 (2022). 10.3389/fmolb.2021.821506 35118120 PMC8804232

[15] W. Dong , H. Mead , L. Tian , J. G. Park , J. I. Garcia , S. Jaramillo , T. Barr , D. S. Kollath , V. K. Coyne , N. E. Stone , A. Jones , J. Zhang , A. Li , L. S. Wang , M. Milanes-Yearsley , J. B. Torrelles , L. Martinez-Sobrido , P. S. Keim , B. M. Barker , M. A. Caligiuri , J. Yu , The K18-Human ACE2 Transgenic Mouse Model Recapitulates Non-severe and Severe COVID-19 in Response to an Infectious Dose of the SARS-CoV-2 Virus. J. Virol. 96, e0096421 (2022). 10.1128/JVI.00964-21 34668775 PMC8754221

[16] P. J. Halfmann , S. Iida , K. Iwatsuki-Horimoto , T. Maemura , M. Kiso , S. M. Scheaffer , T. L. Darling , A. Joshi , S. Loeber , G. Singh , S. L. Foster , B. Ying , J. B. Case , Z. Chong , B. Whitener , J. Moliva , K. Floyd , M. Ujie , N. Nakajima , M. Ito , R. Wright , R. Uraki , P. Warang , M. Gagne , R. Li , Y. Sakai-Tagawa , Y. Liu , D. Larson , J. E. Osorio , J. P. Hernandez-Ortiz , A. R. Henry , K. Ciuoderis , K. R. Florek , M. Patel , A. Odle , L. R. Wong , A. C. Bateman , Z. Wang , V. V. Edara , Z. Chong , J. Franks , T. Jeevan , T. Fabrizio , J. DeBeauchamp , L. Kercher , P. Seiler , A. S. Gonzalez-Reiche , E. M. Sordillo , L. A. Chang , H. van Bakel , V. Simon , D. C. Douek , N. J. Sullivan , L. B. Thackray , H. Ueki , S. Yamayoshi , M. Imai , S. Perlman , R. J. Webby , R. A. Seder , M. S. Suthar , A. García-Sastre , M. Schotsaert , T. Suzuki , A. C. M. Boon , M. S. Diamond , Y. Kawaoka ; Consortium Mount Sinai Pathogen Surveillance (PSP) study group , SARS-CoV-2 Omicron virus causes attenuated disease in mice and hamsters. Nature 603, 687–692 (2022). 10.1038/s41586-022-04441-6 35062015 PMC8942849

[17] D. Mannar , J. W. Saville , X. Zhu , S. S. Srivastava , A. M. Berezuk , K. S. Tuttle , A. C. Marquez , I. Sekirov , S. Subramaniam , SARS-CoV-2 Omicron variant: Antibody evasion and cryo-EM structure of spike protein-ACE2 complex. Science 375, 760–764 (2022). 10.1126/science.abn7760 35050643 PMC9799367

[18] P. Han , L. Li , S. Liu , Q. Wang , D. Zhang , Z. Xu , P. Han , X. Li , Q. Peng , C. Su , B. Huang , D. Li , R. Zhang , M. Tian , L. Fu , Y. Gao , X. Zhao , K. Liu , J. Qi , G. F. Gao , P. Wang , Receptor binding and complex structures of human ACE2 to spike RBD from omicron and delta SARS-CoV-2. Cell 185, 630–640.e10 (2022). 10.1016/j.cell.2022.01.001 35093192 PMC8733278

[19] Z. Cui , P. Liu , N. Wang , L. Wang , K. Fan , Q. Zhu , K. Wang , R. Chen , R. Feng , Z. Jia , M. Yang , G. Xu , B. Zhu , W. Fu , T. Chu , L. Feng , Y. Wang , X. Pei , P. Yang , X. S. Xie , L. Cao , Y. Cao , X. Wang , Structural and functional characterizations of infectivity and immune evasion of SARS-CoV-2 Omicron. Cell 185, 860–871.e13 (2022). 10.1016/j.cell.2022.01.019 35120603 PMC8786603

[20] K. K. Chan , T. J. C. Tan , K. K. Narayanan , E. Procko , An engineered decoy receptor for SARS-CoV-2 broadly binds protein S sequence variants. Sci. Adv. 7, eabf1738 (2021). 10.1126/sciadv.abf1738 33597251 PMC7888922

[21] T. N. Starr , A. J. Greaney , S. K. Hilton , D. Ellis , K. H. D. Crawford , A. S. Dingens , M. J. Navarro , J. E. Bowen , M. A. Tortorici , A. C. Walls , N. P. King , D. Veesler , J. D. Bloom , Deep Mutational Scanning of SARS-CoV-2 Receptor Binding Domain Reveals Constraints on Folding and ACE2 Binding. Cell 182, 1295–1310.e20 (2020). 10.1016/j.cell.2020.08.012 32841599 PMC7418704

[22] A. Hoshino , W. J. Wang , S. Wada , C. McDermott-Roe , C. S. Evans , B. Gosis , M. P. Morley , K. S. Rathi , J. Li , K. Li , S. Yang , M. J. McManus , C. Bowman , P. Potluri , M. Levin , S. Damrauer , D. C. Wallace , E. L. F. Holzbaur , Z. Arany , The ADP/ATP translocase drives mitophagy independent of nucleotide exchange. Nature 575, 375–379 (2019). 10.1038/s41586-019-1667-4 31618756 PMC6858570

[23] H. K. Haddox , A. S. Dingens , S. K. Hilton , J. Overbaugh , J. D. Bloom , Mapping mutational effects along the evolutionary landscape of HIV envelope. eLife 7, e34420 (2018). 10.7554/eLife.34420 29590010 PMC5910023

[24] J. M. Lee , J. Huddleston , M. B. Doud , K. A. Hooper , N. C. Wu , T. Bedford , J. D. Bloom , Deep mutational scanning of hemagglutinin helps predict evolutionary fates of human H3N2 influenza variants. Proc. Natl. Acad. Sci. U.S.A. 115, E8276–E8285 (2018). 10.1073/pnas.1806133115 30104379 PMC6126756

[25] A. Baum , B. O. Fulton , E. Wloga , R. Copin , K. E. Pascal , V. Russo , S. Giordano , K. Lanza , N. Negron , M. Ni , Y. Wei , G. S. Atwal , A. J. Murphy , N. Stahl , G. D. Yancopoulos , C. A. Kyratsous , Antibody cocktail to SARS-CoV-2 spike protein prevents rapid mutational escape seen with individual antibodies. Science 369, 1014–1018 (2020). 10.1126/science.abd0831 32540904 PMC7299283

[26] T. N. Starr , A. J. Greaney , A. Addetia , W. W. Hannon , M. C. Choudhary , A. S. Dingens , J. Z. Li , J. D. Bloom , Prospective mapping of viral mutations that escape antibodies used to treat COVID-19. Science 371, 850–854 (2021). 10.1126/science.abf9302 33495308 PMC7963219

[27] K. Uriu , I. Kimura , K. Shirakawa , A. Takaori-Kondo , T. A. Nakada , A. Kaneda , S. Nakagawa , K. Sato ; Genotype to Phenotype Japan (G2P-Japan) Consortium , Neutralization of the SARS-CoV-2 Mu Variant by Convalescent and Vaccine Serum. N. Engl. J. Med. 385, 2397–2399 (2021). 10.1056/NEJMc2114706 34731554 PMC8609602

[28] Q. Li , J. Nie , J. Wu , L. Zhang , R. Ding , H. Wang , Y. Zhang , T. Li , S. Liu , M. Zhang , C. Zhao , H. Liu , L. Nie , H. Qin , M. Wang , Q. Lu , X. Li , J. Liu , H. Liang , Y. Shi , Y. Shen , L. Xie , L. Zhang , X. Qu , W. Xu , W. Huang , Y. Wang , SARS-CoV-2 501Y.V2 variants lack higher infectivity but do have immune escape. Cell 184, 2362–2371.e9 (2021). 10.1016/j.cell.2021.02.042 33735608 PMC7901273

[29] W. Dejnirattisai , J. Huo , D. Zhou , J. Zahradník , P. Supasa , C. Liu , H. M. E. Duyvesteyn , H. M. Ginn , A. J. Mentzer , A. Tuekprakhon , R. Nutalai , B. Wang , A. Dijokaite , S. Khan , O. Avinoam , M. Bahar , D. Skelly , S. Adele , S. A. Johnson , A. Amini , T. G. Ritter , C. Mason , C. Dold , D. Pan , S. Assadi , A. Bellass , N. Omo-Dare , D. Koeckerling , A. Flaxman , D. Jenkin , P. K. Aley , M. Voysey , S. A. Costa Clemens , F. G. Naveca , V. Nascimento , F. Nascimento , C. Fernandes da Costa , P. C. Resende , A. Pauvolid-Correa , M. M. Siqueira , V. Baillie , N. Serafin , G. Kwatra , K. Da Silva , S. A. Madhi , M. C. Nunes , T. Malik , P. J. M. Openshaw , J. K. Baillie , M. G. Semple , A. R. Townsend , K. A. Huang , T. K. Tan , M. W. Carroll , P. Klenerman , E. Barnes , S. J. Dunachie , B. Constantinides , H. Webster , D. Crook , A. J. Pollard , T. Lambe , N. G. Paterson , M. A. Williams , D. R. Hall , E. E. Fry , J. Mongkolsapaya , J. Ren , G. Schreiber , D. I. Stuart , G. R. Screaton ; OPTIC Consortium; ISARIC4C Consortium , SARS-CoV-2 Omicron-B.1.1.529 leads to widespread escape from neutralizing antibody responses. Cell 185, 467–484.e15 (2022). 10.1016/j.cell.2021.12.046 35081335 PMC8723827

[30] W. Dejnirattisai , R. H. Shaw , P. Supasa , C. Liu , A. S. Stuart , A. J. Pollard , X. Liu , T. Lambe , D. Crook , D. I. Stuart , J. Mongkolsapaya , J. S. Nguyen-Van-Tam , M. D. Snape , G. R. Screaton ; Com-COV2 study group , Reduced neutralisation of SARS-CoV-2 omicron B.1.1.529 variant by post-immunisation serum. Lancet 399, 234–236 (2022). 10.1016/S0140-6736(21)02844-0 34942101 PMC8687667

[31] Y. Cao , J. Wang , F. Jian , T. Xiao , W. Song , A. Yisimayi , W. Huang , Q. Li , P. Wang , R. An , J. Wang , Y. Wang , X. Niu , S. Yang , H. Liang , H. Sun , T. Li , Y. Yu , Q. Cui , S. Liu , X. Yang , S. Du , Z. Zhang , X. Hao , F. Shao , R. Jin , X. Wang , J. Xiao , Y. Wang , X. S. Xie , Omicron escapes the majority of existing SARS-CoV-2 neutralizing antibodies. Nature 602, 657–663 (2022). 10.1038/s41586-021-04385-3 35016194 PMC8866119

[32] A. Rössler , L. Riepler , D. Bante , D. von Laer , J. Kimpel , SARS-CoV-2 Omicron Variant Neutralization in Serum from Vaccinated and Convalescent Persons. N. Engl. J. Med. 386, 698–700 (2022). 10.1056/NEJMc2119236 35021005 PMC8781314

[33] K. R. McCarthy , L. J. Rennick , S. Nambulli , L. R. Robinson-McCarthy , W. G. Bain , G. Haidar , W. P. Duprex , Recurrent deletions in the SARS-CoV-2 spike glycoprotein drive antibody escape. Science 371, 1139–1142 (2021). 10.1126/science.abf6950 33536258 PMC7971772

[34] A. J. Greaney , A. N. Loes , L. E. Gentles , K. H. D. Crawford , T. N. Starr , K. D. Malone , H. Y. Chu , J. D. Bloom , Antibodies elicited by mRNA-1273 vaccination bind more broadly to the receptor binding domain than do those from SARS-CoV-2 infection. Sci. Transl. Med. 13, eabi9915 (2021). 10.1126/scitranslmed.abi9915 34103407 PMC8369496

[35] W. Yao , D. Ma , H. Wang , X. Tang , C. Du , H. Pan , C. Li , H. Lin , M. Farzan , J. Zhao , Y. Li , G. Zhong , Effect of SARS-CoV-2 spike mutations on animal ACE2 usage and in vitro neutralization sensitivity. bioRxiv, 2021.2001.2027.428353 (2021). 10.1101/2021.01.27.428353

[36] N. Saitou , M. Nei , The neighbor-joining method: A new method for reconstructing phylogenetic trees. Mol. Biol. Evol. 4, 406–425 (1987). 3447015 10.1093/oxfordjournals.molbev.a040454

[37] K. Katoh , D. M. Standley , MAFFT multiple sequence alignment software version 7: Improvements in performance and usability. Mol. Biol. Evol. 30, 772–780 (2013). 10.1093/molbev/mst010 23329690 PMC3603318

[38] T. Giroglou , J. Cinatl Jr ., H. Rabenau , C. Drosten , H. Schwalbe , H. W. Doerr , D. von Laer , Retroviral vectors pseudotyped with severe acute respiratory syndrome coronavirus S protein. J. Virol. 78, 9007–9015 (2004). 10.1128/JVI.78.17.9007-9015.2004 15308697 PMC506966

[39] M. A. Tortorici , N. Czudnochowski , T. N. Starr , R. Marzi , A. C. Walls , F. Zatta , J. E. Bowen , S. Jaconi , J. Di Iulio , Z. Wang , A. De Marco , S. K. Zepeda , D. Pinto , Z. Liu , M. Beltramello , I. Bartha , M. P. Housley , F. A. Lempp , L. E. Rosen , E. Dellota Jr ., H. Kaiser , M. Montiel-Ruiz , J. Zhou , A. Addetia , B. Guarino , K. Culap , N. Sprugasci , C. Saliba , E. Vetti , I. Giacchetto-Sasselli , C. S. Fregni , R. Abdelnabi , S. C. Foo , C. Havenar-Daughton , M. A. Schmid , F. Benigni , E. Cameroni , J. Neyts , A. Telenti , H. W. Virgin , S. P. J. Whelan , G. Snell , J. D. Bloom , D. Corti , D. Veesler , M. S. Pizzuto , Broad sarbecovirus neutralization by a human monoclonal antibody. Nature 597, 103–108 (2021). 10.1038/s41586-021-03817-4 34280951 PMC9341430

[40] S. Ozono , Y. Zhang , M. Tobiume , S. Kishigami , K. Tokunaga , Super-rapid quantitation of the production of HIV-1 harboring a luminescent peptide tag. J. Biol. Chem. 295, 13023–13030 (2020). 10.1074/jbc.RA120.013887 32719008 PMC7489901

[41] C. Shema Mugisha , H. R. Vuong , M. Puray-Chavez , A. L. Bailey , J. M. Fox , R. E. Chen , A. W. Wessel , J. M. Scott , H. H. Harastani , A. C. M. Boon , H. Shin , S. B. Kutluay , A Simplified Quantitative Real-Time PCR Assay for Monitoring SARS-CoV-2 Growth in Cell Culture. MSphere 5, e00658-20 (2020). 10.1128/mSphere.00658-20 32878932 PMC7471006

