# Supplementary material for: An engineered ACE2 decoy neutralizes the SARS-CoV-2 Omicron variant and confers protection against infection in vivo
Source: Sci Transl Med. 2022 Apr 26:eabn7737. doi: 10.1126/scitranslmed.abn7737 (PMC9097879; doi:10.1126/scitranslmed.abn7737)
Supplement: Supplementary file 1 — Figs. S1 to S10 [file scitranslmed.abn7737_sm.pdf]

Science Translational Medicine
AAAS

Supplementary Materials for

An engineered ACE2 decoy neutralizes the SARS-CoV-2 Omicron variant and confers protection against infection in vivo

Nariko Ikemura *et al.*

Corresponding authors: Atsushi Hoshino, a-hoshi@koto.kpu-m.ac.jp; Junichi Takagi, takagi@protein.osaka-u.ac.jp; Toru Okamoto, toru@biken.osaka-u.ac.jp; Daron M Standley, standley@biken.osaka-u.ac.jp

DOI: 10.1126/scitranslmed.abn7737

The PDF file includes:

Figs. S1 to S10

Other Supplementary Material for this manuscript includes the following:

MDAR Reproducibility Checklist
Data file S1

Supplementary Materials

The PDF file includes:

Figs. S1 to S10

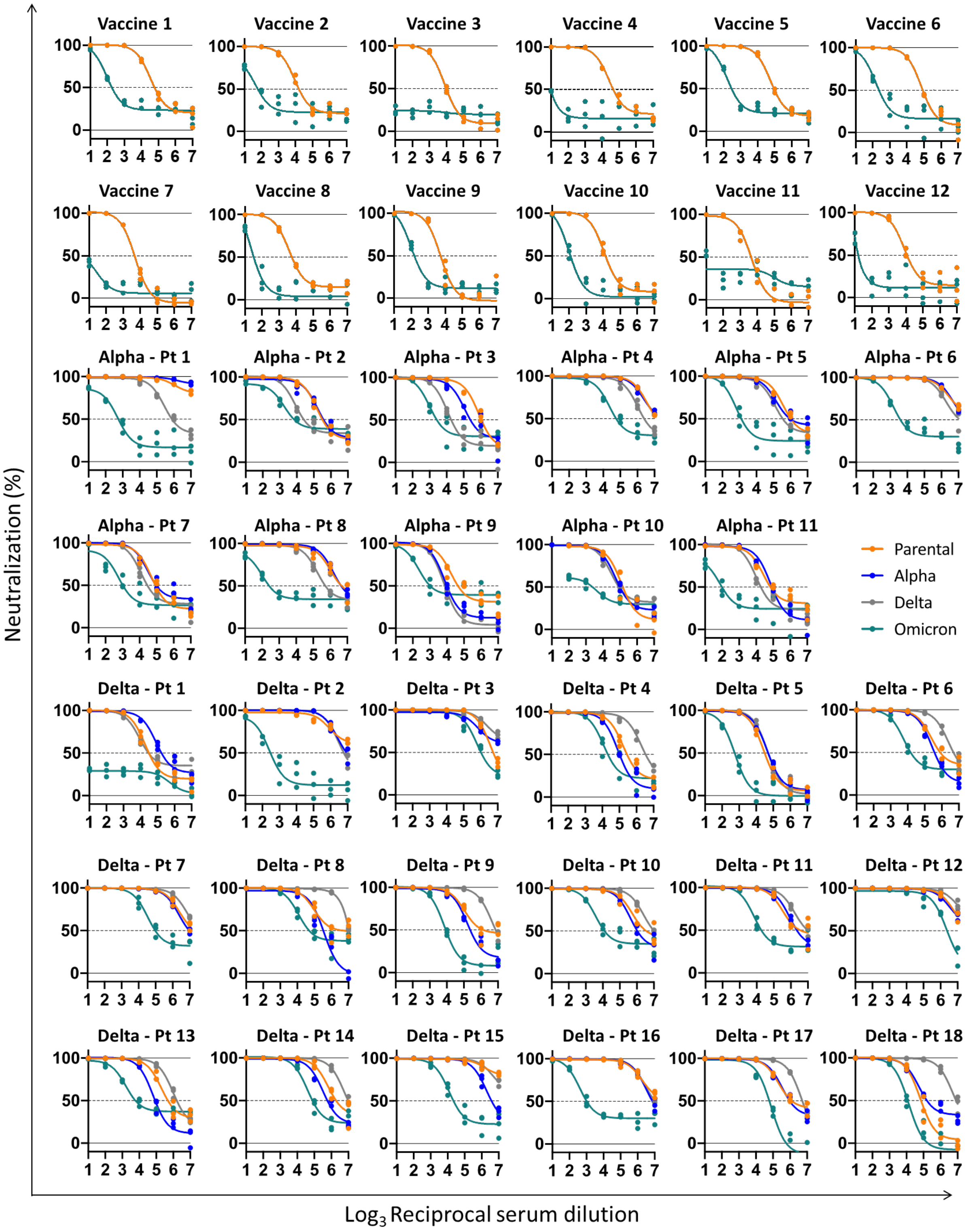

Fig. S1. Neutralization assay with SARS-CoV-2 spike protein-expressing pseudoviruses. Data are shown for individual vaccine recipients (top) as well as patients (Pt) infected with the alpha or delta variants. Data are summarized in Fig. 1A to 1C. Neutralization of the parental (D614G) pseudovirus (orange), Alpha variant (blue), Delta variant (gray) and Omicron variant (green) was analyzed in 293T cells expressing angiotensin converting enzyme 2 (ACE2). n = 3 technical replicates.

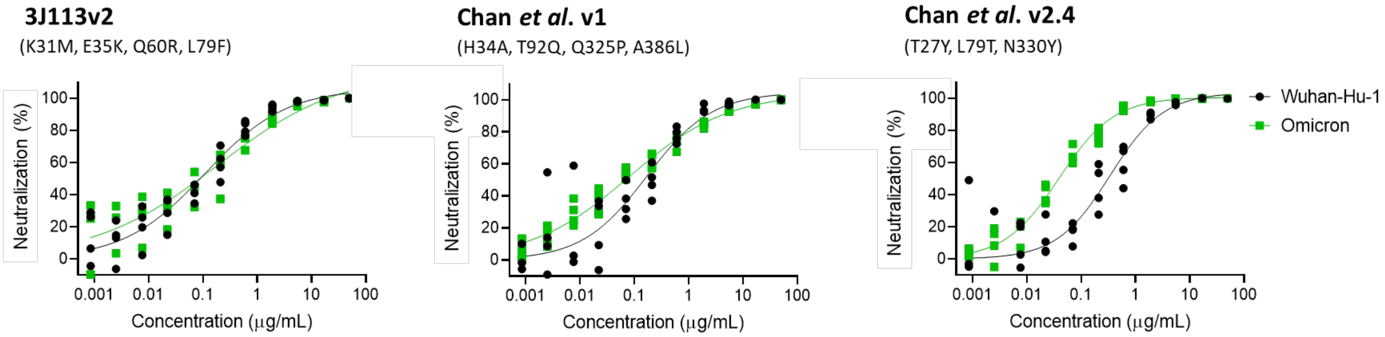

Fig. S2. Neutralization assay for engineered ACE2s. Neutralization efficacy against the Omicron variant is shown for 3J113v2 from our previous study and v1 and v2.4 in Chan *et al. (7)*; these three different ACE2-Fc molecules (615 amino acids, without the collectrin domain) inhibited Omicron pseudovirus infection of 293T/ACE2 cells. n = 4 technical replicates. ACE2 mutations are indicated.

```
    Wuhan RVQPTESIVRFPNITNLCPFGEVFNATRFASVYAWNRKRISNCVADYSVLYNSASFSTFKCYGVSPTKLNDLCFTNVYAD
    Delta ................................................................................
  Epsilon ................................................................................
    Alpha ................................................................................
      Mu1 ...........................K....................................................
     Beta ................................................................................
    Gamma ................................................................................
   Lambda ................................................................................
  Omicron ....................D...............................L.P.F.......................
   PG-GD1 ...........................T.........................T..........................
   RaTG13 .....D.....................T.........................T..........................
    PG-GX .....I....................SK.........................T..........................
   Rs4231 ..A.SKEV...................T.P.....E.................T...........A........S.....
 RsSHC014 ..A.SKEV...................T.P.....E.................T...........A........S.....
     WIV1 ..A.SKEV...................T.P.....E.................T...........A........S.....
    SARS1 ..V.SGDV...................K.P.....E..K..............TF..........A........S.....

    Wuhan SFVIRGDEVRQIAPGQTGKIADYNYKLPDDFTGCVIAWNSNNLDSKVGGNYNYLYRLFRKSNLKPFERDISTEIYQAGST
    Delta .....................................................R.........................K
  Epsilon .....................................................R..........................
    Alpha ................................................................................
      Mu1 ................................................................................
     Beta ..................N.............................................................
    Gamma ..................T.............................................................
   Lambda .....................................................Q..........................
  Omicron ..................N......................K.....S..............................NK
   PG-GD1 ...V..............R.............................................................
   RaTG13 ....T...................................KHI.A.E...F.........A..................K
    PG-GX ...VK.............V.....................VKQ.ALT..--..........K..................
   Rs4231 ...VK..D..........V............L...L...T.SK..STS........WV.R.K.N.Y...L.ND..SP.GQ
 RsSHC014 ...VK..D..........V............L...L...T.SK..STS........WV.R.K.N.Y...L.ND..SP.GQ
     WIV1 ...VK..D..........V................L...TR.I.ATQT.....K..SL.HGK.R.......NVPFSPDGK
    SARS1 ...VK..D..........V............M...L...TR.I.ATST.....K..YL.HGK.R.......NVPFSPDGK

    Wuhan PCNGVEGFNCYFPLQSYGFQPTNGVGYQPYRVVVLSFELLHAPATVCGPKKSTNLVKNKCVNF
    Delta ...............................................................
  Epsilon ...............................................................
    Alpha ......................Y........................................
      Mu1 .....K................Y........................................
     Beta .....K................Y........................................
    Gamma .....K................Y........................................
   Lambda ...........S...................................................
  Omicron .....A........R..S.R..Y...H....................................
   PG-GD1 ...................H....................N.........Q............
   RaTG13 ....QT.L...Y..YR...Y..D...H.............N......................
    PG-GX ....QV.L...Y..ER...H..T..N...F..........NG........L..T...D.....
   Rs4231 S.SA-I.P...N..RP...FT.A...H.............N.........L..D.I..Q....
 RsSHC014 S.SA-V.P...N..RP...FT.A...H.............N.........L..D.I..Q....
     WIV1 ..TP-PA....W..ND...YI...I...............N.........L..D.I..Q....
    SARS1 ..TP-PAL...W..ND...YT.T.I...............N.........L..D.I..Q....
```

Fig.S3 Alignment of amino acid sequences of sarbecovirus receptor binding domains (RBDs). ACE2 contacts are highlighted by cyan based on the crystal structure (PDB: 6M0J). Omicron is highlighted in red.

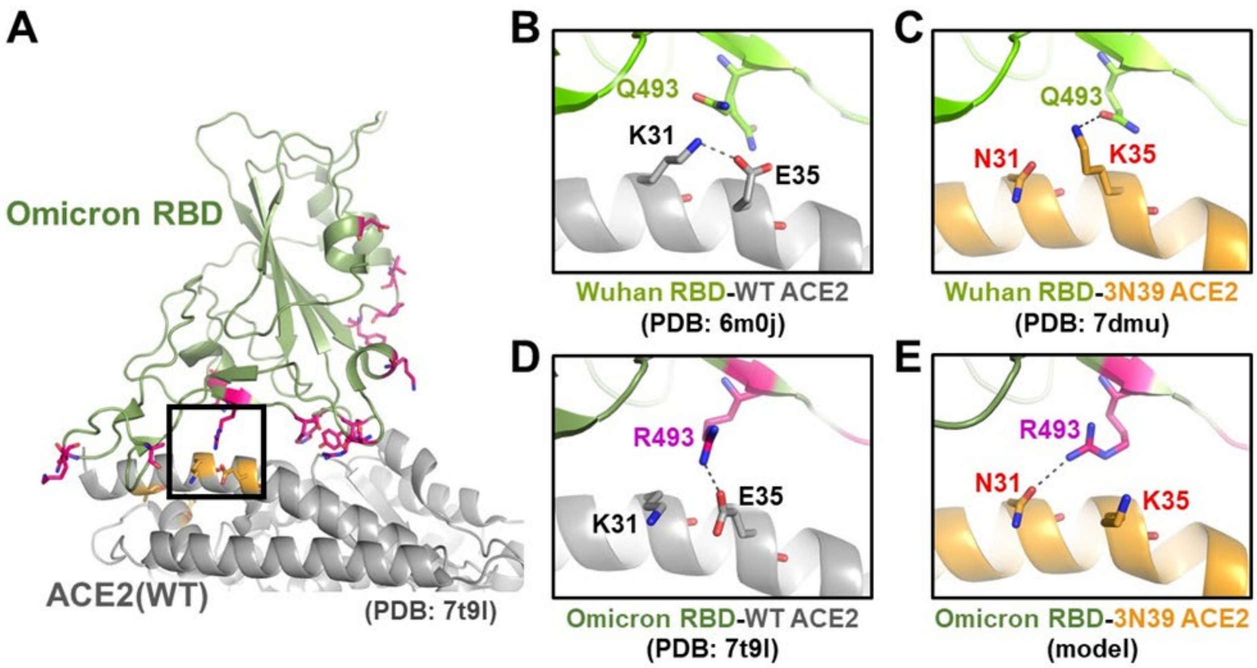

Fig. S4. Differences in the interaction mode of Wuhan RBD and Omicron RBD to ACE2 mutant. (**A**) Cryo-electron microscopy structure of the wild type (WT) ACE2-Omicron RBD complex (Manner *et al*., PDB ID: 7t9i). Residues mutated in Omicron are shown as magenta stick models. The ACE2 residues mutated in 3N39v4 (only A25, K31, E35, and T92 are close to the interface and visible in this view) are shown in pale orange. (**B to E**) Close-up views of the interface between (B) Wuhan RBD and WT ACE2 (PDB ID: 6m0j), (C) Wuhan RBD-3N39 and ACE2 (PDB ID: 7dmu), (D) Omicron RBD and WT ACE2 (PDB ID: 7t9l), and (E) Omicron RBD and 3N39 ACE2 (simulated model) are shown. Potential hydrogen-bonding and salt-bridge interactions are indicated by dashed lines.

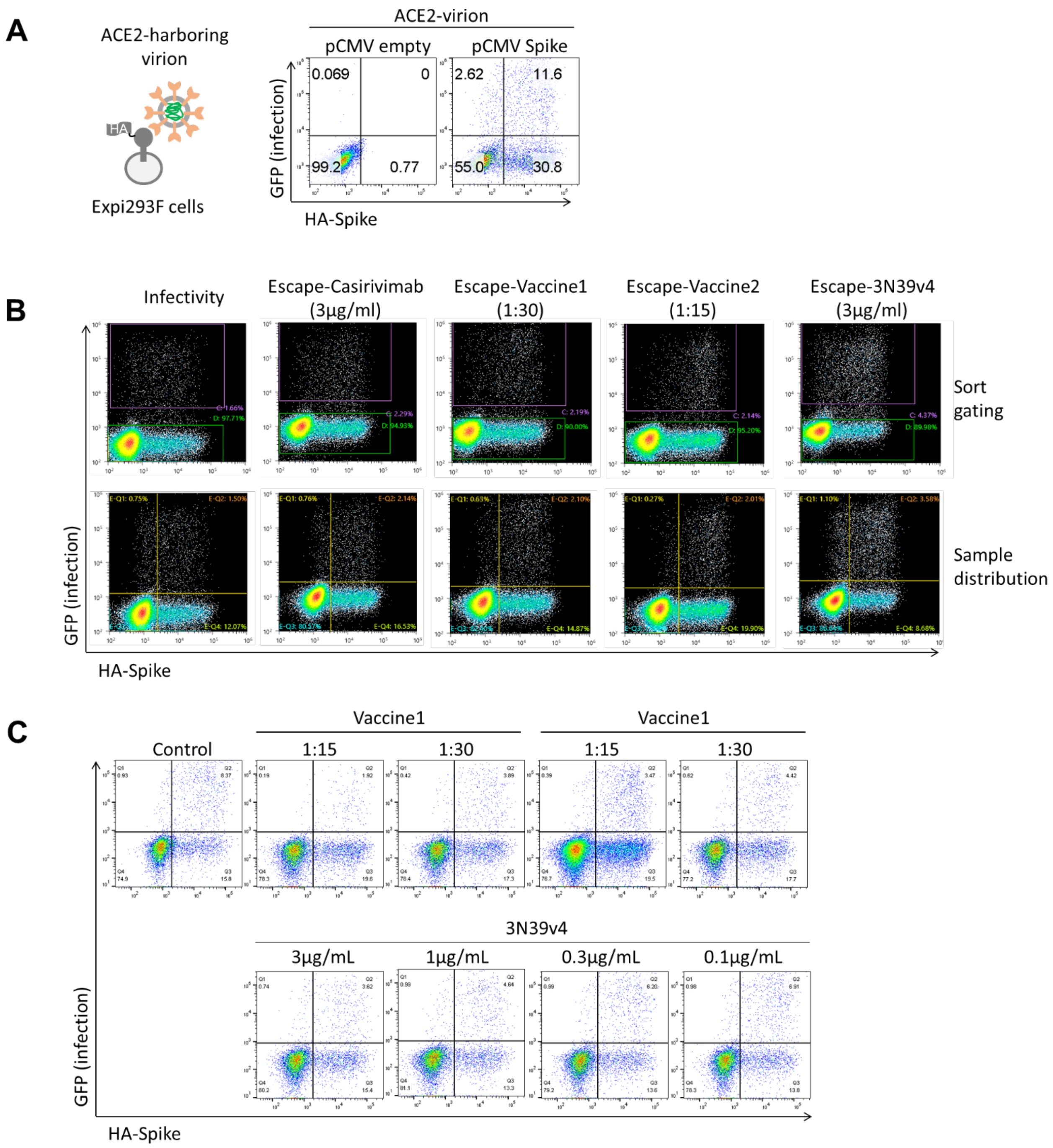

Fig. S5. Deep mutational scanning (DMS) for infectivity and escape using spike protein-expressing cells and ACE2-harboring virions. (**A**) Flow cytometry results showing that only the spike protein-expressing cells are infected by ACE2-virions. HA, hemagglutinin; pCMV, cytomegalovirus; GFP, green fluorescent protein. (**B**) Upper panels are the gate setting to sort infected cells (gate C) and non-infected cells (gate D). Lower panels show the proportion of each population. (**C**) Titrations were conducted to determine the optimal dilution of serum and concentration of engineered ACE2. DMS for infectivity and escape phenotypes was performed in the setting of about 3% infected cells among about 15% spike protein-expressing cells.

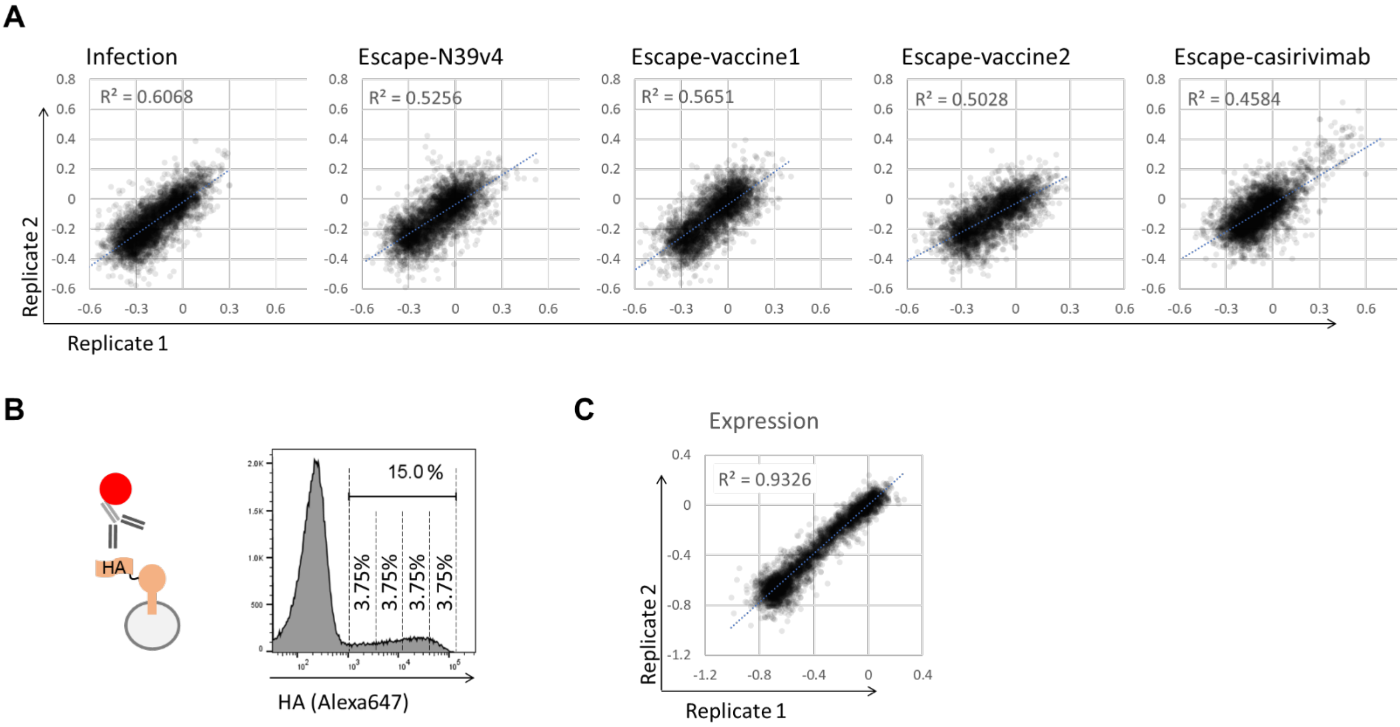

Fig. S6. Reproducibility of each deep mutational scan for infectivity and escape from neutralizing agents. (**A**) The correlation in mutation effects on the alteration of infectivity or escape from each neutralizing agent across replicates is shown. (**B**) The gating for fluorescence activated cell sorting (FACS) to perform DMS is shown. Cells were gated on spike protein expression based on the staining of the HA tag. Among the 15% of HA-positive cells, the top 25% and bottom 25% of cells were sorted. (**C**) The correlation in mutation effects on the alteration of spike protein expression across replicates are shown.

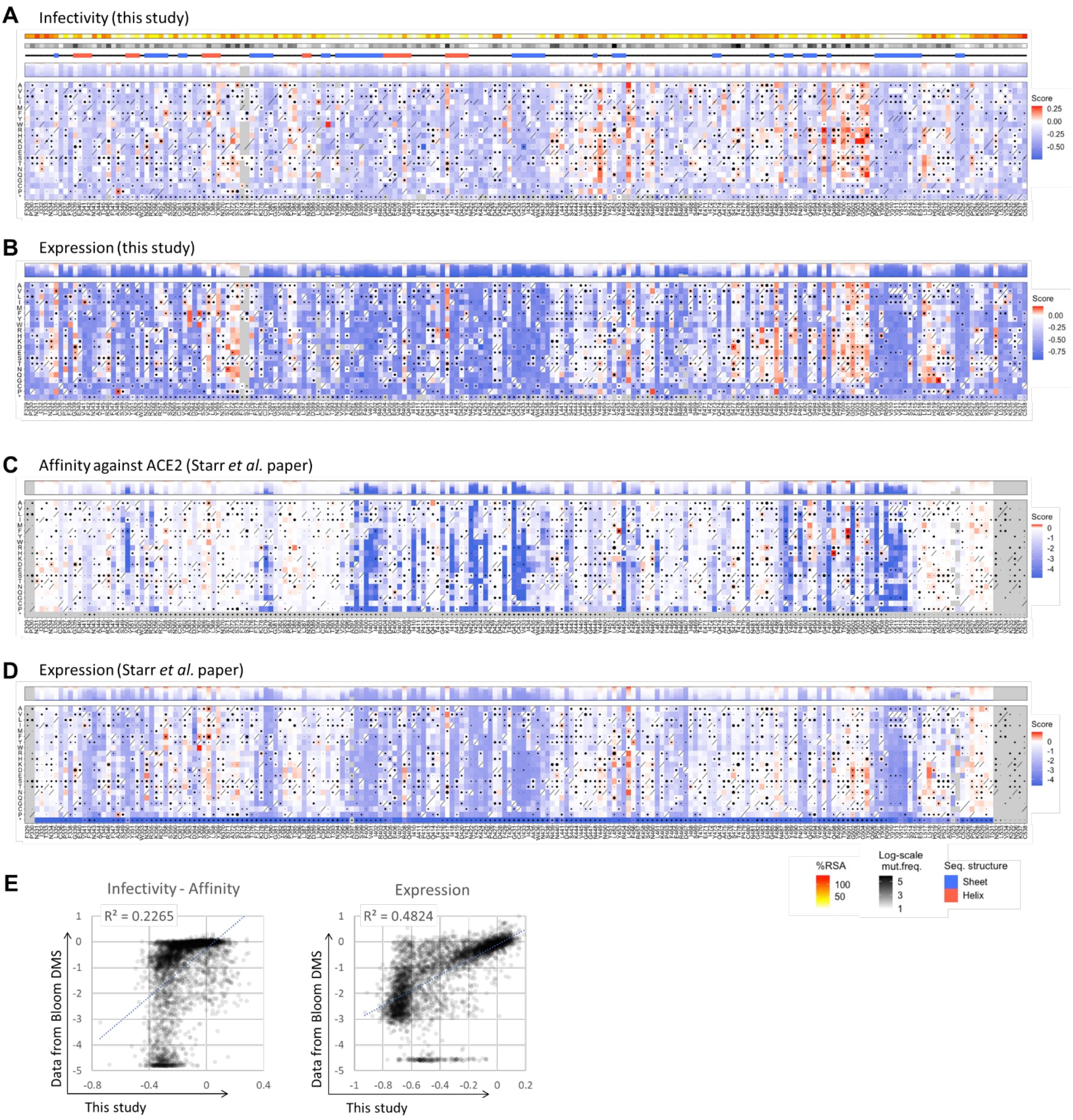

Fig. S7. Sequence-to-Phenotype Maps of the whole RBD for infectivity and expression. (**A and B**) Heatmaps demonstrating how all single mutations affects infectivity (A) and spike expression (B) in this study are shown. (**C and D**) Heatmaps of DMS retrieved from Starr *et al.* *(21)* for affinity against ACE2 (C) and RBD expression (D) are shown. (**E**) Scatter plot showing the correlation between this study and the DMS results from Starr *et al*. The left panel compares infectivity of this study and affinity of Starr *et al.* DMS. Right panel compares expression values for the two studies. Squares are colored by mutational effect

according to scale bars on the right, with blue indicating deleterious mutations. Squares with a diagonal line through them indicate the original Wuhan strain amino acid. Black dot size reflects the frequency in the virus genome sequence according to GISAID database as 17th Dec 2021. %RSA is the relative solvent accessibility in the crystal structure of ACE2-Spike protein complex.

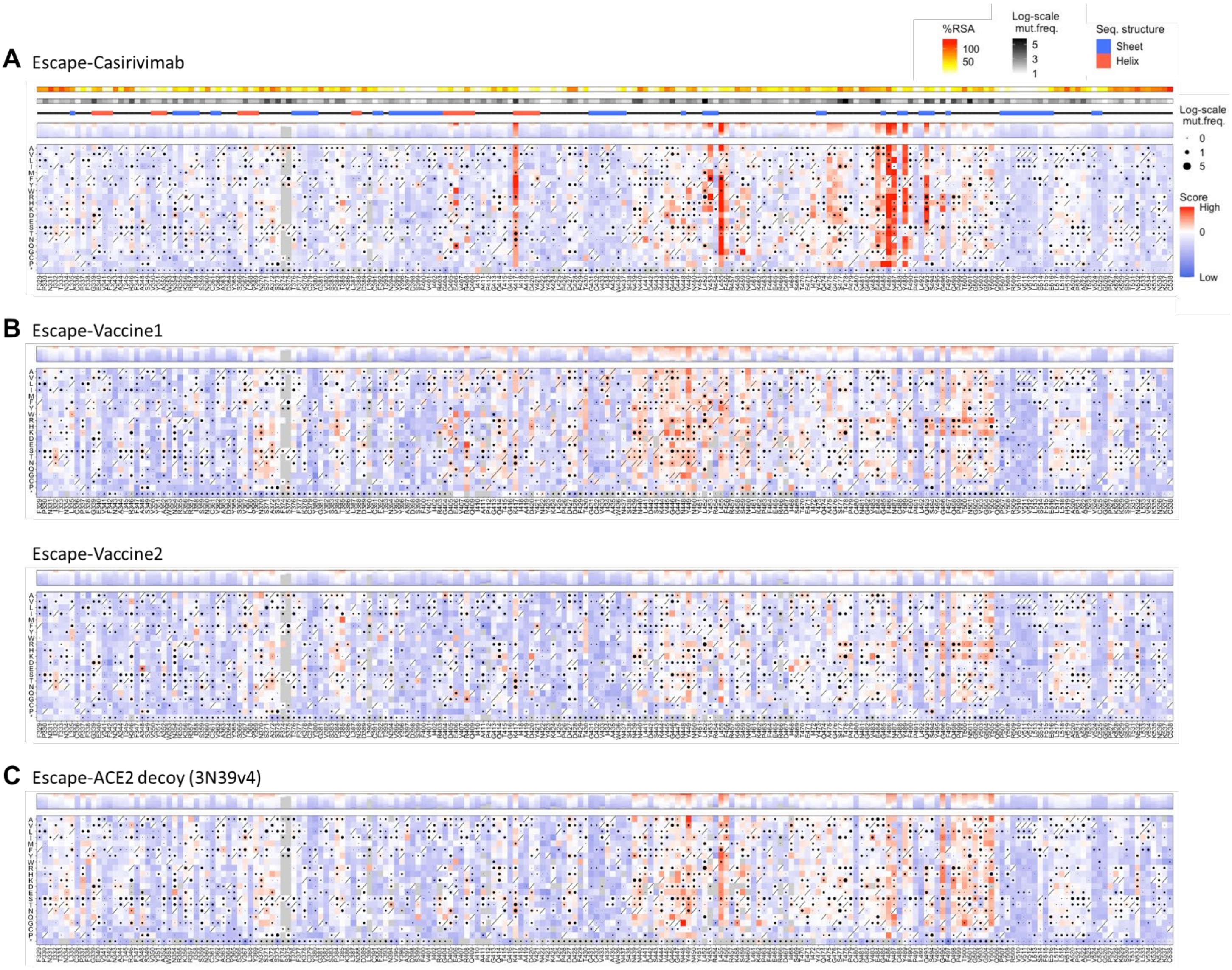

Fig. S8. Sequence-to-Phenotype Maps of the whole RBD for escape. (**A** to **C**) Heatmaps are shown demonstrating how all single mutations affects escape from casirivimab (A), vaccinated serum samples (B) and engineered ACE2 (C). Squares are colored by mutational effect according to scale bar on the right. Squares with a diagonal line through them indicate the original Wuhan strain amino acid. Black dot size reflects the frequency in the virus genome sequence according to GISAID database as 17th Dec 2021. %RSA is the relative solvent accessibility in the crystal structure of ACE2-Spike protein complex. AA, amino acid.

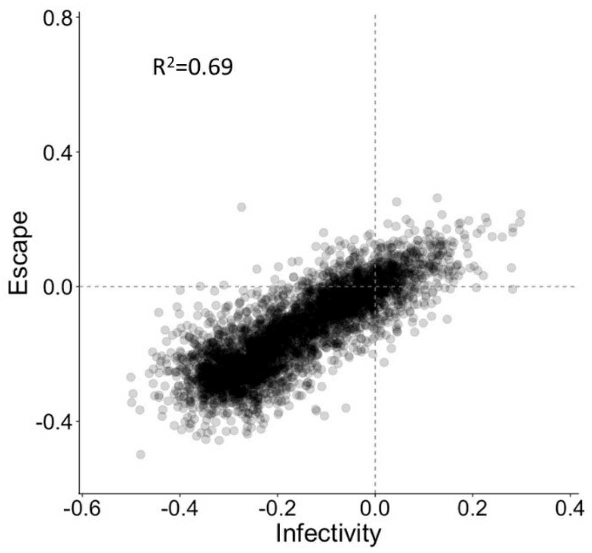

Fig. S9. Reproducibility of correlation between infectivity and escape in the vaccinated serum samples. The correlation in mutation effects on the alteration of infectivity or escape from the neutralization are shown for serum samples from vaccinated individuals.

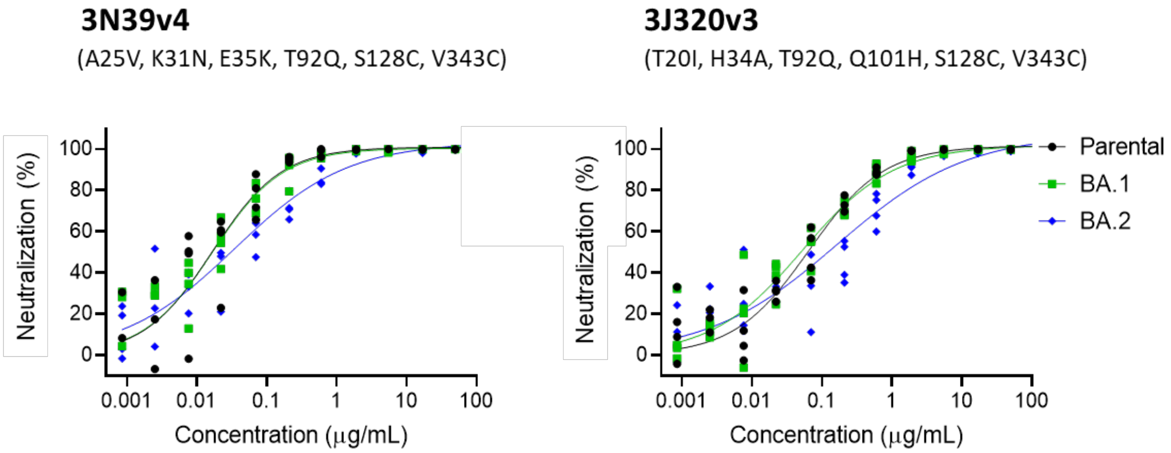

Fig. S10. Neutralization assay for engineered ACE2 proteins against the Omicron subvariant BA.2. Neutralization efficacy of 3N39v4 and 3J320v3 against parental virus (D614G mutation) and Omicron subvariants BA.1 and BA.2 pseudoviruses were analyzed in 293T/ACE2 cells. n = 4 technical replicates. ACE2 mutations are indicated.
